# Disease Localization in Multilayer Networks

**DOI:** 10.1103/PhysRevX.7.011014

**Published:** 2017-02-02

**Authors:** Guilherme Ferraz de Arruda, Emanuele Cozzo, Tiago P. Peixoto, Francisco A. Rodrigues, Yamir Moreno

**Affiliations:** ^1^ Departamento de Matemática Aplicada e Estatística, Instituto de Ciências Matemáticas e de Computação, Universidade de São Paulo—Campus de São Carlos, Caixa Postal 668, 13560-970 São Carlos, São Paulo, Brazil; ^2^ Institute for Biocomputation and Physics of Complex Systems (BIFI), University of Zaragoza, Zaragoza 50009, Spain; ^3^ Department of Theoretical Physics, University of Zaragoza, Zaragoza 50009, Spain; ^4^ Department of Mathematical Sciences and Centre for Networks and Collective Behaviour, University of Bath, Claverton Down, Bath BA2 7AY, United Kingdom; ^5^ ISI Foundation, Via Alassio 11/c, 10126 Torino, Italy

## Abstract

We present a continuous formulation of epidemic spreading on multilayer networks using a tensorial representation, extending the models of monoplex networks to this context. We derive analytical expressions for the epidemic threshold of the susceptible-infected-susceptible (SIS) and susceptible-infected-recovered dynamics, as well as upper and lower bounds for the disease prevalence in the steady state for the SIS scenario. Using the quasistationary state method, we numerically show the existence of disease localization and the emergence of two or more susceptibility peaks, which are characterized analytically and numerically through the inverse participation ratio. At variance with what is observed in single-layer networks, we show that disease localization takes place on the layers and not on the nodes of a given layer. Furthermore, when mapping the critical dynamics to an eigenvalue problem, we observe a characteristic transition in the eigenvalue spectra of the supra-contact tensor as a function of the ratio of two spreading rates: If the rate at which the disease spreads within a layer is comparable to the spreading rate across layers, the individual spectra of each layer merge with the coupling between layers. Finally, we report on an interesting phenomenon, the barrier effect; i.e., for a three-layer configuration, when the layer with the lowest eigenvalue is located at the center of the line, it can effectively act as a barrier to the disease. The formalism introduced here provides a unifying mathematical approach to disease contagion in multiplex systems, opening new possibilities for the study of spreading processes.

## Summary

Networks are all around. They describe the flow of information, the movement of people and goods via multiple modes of transportation, and the spread of disease across interconnected populations. Traditionally, networks have been studied as if they were a single layer, which flattens out hierarchies such as social circles. Multilayer networks, which consider each of those circles as a layer, are more accurate descriptions of real-world networks and their use can have deep implications for understanding the dynamics of the system. Using the spread of disease as a model, we have developed a mathematical framework that accounts for the multilayer structure, and we have identified several behaviors that emerge from this analysis.

The framework relies on tensors, mathematical objects that allow us to represent multidimensional data in a compact way. Through mathematical analysis and numerical simulations, we find a number of interesting features such as the existence of multiple epidemic thresholds and transmission rates beyond which the number of individuals that catch a disease is non-negligible. We also show the existence of disease localization, a scenario in which the disease cannot escape a layer and jump to another.

Our work provides a unifying mathematical approach to studying disease transmission among multilayered populations. There are still many aspects to investigate such as how to use these results to help contain an epidemic as well as how the picture changes in more complex scenarios. Disease-like models can also be used to explore other networks such as the propagation of information.

## I. INTRODUCTION

Epidemic-like spreading processes are paradigmatic, as they can describe not only the temporal unfolding and evolution of diseases but also of ideas, information, and rumors in fields as diverse as biological, information, and social sciences [1]. Because of their fundamental nature and simplicity, two particular models have received special attention by the scientific community, the susceptible-infected-susceptible (SIS) and the susceptible-infected-recovered (SIR) models. In both models, an infected individual spreads the disease to its neighbors at a given (spreading) rate, and infected individuals recover at some other rate. The difference between both scenarios lies in the fact that in the SIS case, once recovered, infected individuals can catch the disease again; therefore, they go back to the susceptible state. On the contrary, in the SIR model, recovered individuals are supposed to acquire permanent immunity and do not play any active role in the spreading process anymore. There are many other variations of these two models, including more realistic and intricate compartmental models [1]. However, these two schemes are sufficient to capture the main phenomenology of disease dynamics—and many other contagion-like processes—including the onset of epidemics, while remaining simple.

Originally, the modeling of diseases was confined to homogeneous systems, where any pair of individuals have the same contact probability [2,3]. However, most real-world networks are heterogeneously organized, leading to the reexamination of previous results considering nontrivial patterns among individuals, such as power-law degree distributions [4–6]. In Ref. [7], the authors presented the heterogeneous mean-field approach (HMF), showing that the epidemic threshold tends to zero in the thermodynamic limit on scale-free networks when the characteristic exponent is less than 3. This observation about the role of network organization completely changed our previous understanding of how disease outbreaks should be modeled and controlled, placing the focus of attention not only on new ways to model disease dynamics but also on the incorporation of real contact patterns in the dynamical settings [3,8–11].

Since then, many computational and theoretical frameworks have been proposed, which undoubtedly made the modeling of disease contagion an active area of research and provided new phenomenological insights and accurate methods for the study of real outbreaks. For instance, instead of the HMF approach, one can adopt the quenched mean-field (QMF) method, where a specific network is fixed and the dynamics is modeled in terms of nodal probabilities [12,13]. The results obtained with the latter approach show that the epidemic threshold depends on the inverse of the leading eigenvalue of the adjacency matrix [12,13]; a similar result was also obtained using a discrete Markov chain approach [14]. Other scenarios explored recently include the case of temporal networks [15,16], competing and interacting diseases [17–23], as well as the inclusion of human behavioral responses [24–26].

However, the vast majority of the works so far deal with single-layered networks, despite the fact that many real systems exhibit a large degree of interconnectivity and hence should be modeled as multilayer networks [27]. Such systems represent multimodal, multicategorical, or temporal interactions, such as social relations, the ecosystem formed by different online social networks, or modern transportation systems [27]. Cozzo *et al.* [28] showed that disregarding the multilayer structure can lead to misleading conclusions, missing fundamental aspects of the critical dynamics of spreadinglike processes. Such findings reinforce the importance of a more detailed investigation of contagion processes on multilayer networks. Here, we develop a theoretical and computational framework for the analysis of disease spreading, generalizing the results of Ref. [13] to multilayer networks. A continuous counterpart to the model presented in Ref. [28] is provided in terms of the tensorial notation introduced in Ref. [29]. Our methodology allows for several new results. First, we are able to write down, in a compact form, the equations describing the disease dynamics in a multilayer system. Second, we derive the corresponding epidemic thresholds for the SIS and SIR cases, as well as establish bounds for the prevalence of the disease in the SIS scenario. More importantly for future works, we identify previously unnoticed multiple susceptibility peaks and show that there is disease localization but that it takes place on the layers instead of on the nodes of a given layer. All these findings are traced back to the very topological nature of the system and described in terms of the eigenvalue spectra of the supra-contact tensor and the localization of eigenstates.

The rest of the paper is organized as follows: We first formally define the concept of multilayer network, introducing the tensorial notation. Next, we derive the equations describing the dynamics of the disease for the SIS scheme, calculating the upper and lower bounds for the prevalence of the disease in the steady state, followed by the analytical expression for the epidemic threshold, which is also derived for the SIR model. Furthermore, we use the results in Ref. [30] to define some constraints on the critical point. In addition, we explore the notion of localization of eigenstates, formerly applied on epidemic spreading in Ref. [31], to inspect layerwise disease localization transitions. Finally, we also present results from extensive numerical simulations considering multiplex networks with scale-free and scale-rich structures, computing their respective epidemic thresholds. We present our conclusions in the last section.

## II. CONTINUOUS FORMULATION FOR MULTILAYER EPIDEMIC SPREADING

Multilayer networks have been shown to better describe interdependent systems. Mathematically, they can be described by either generalizing the matrix representation and formalism [27] or by encoding the system’s topology in a tensorial representation, which was recently proposed [29] and first applied to describe a dynamical process in Ref. [32]. Here, we use the latter framework to formulate a continuous time Markov chain model that describes the evolution of an epidemic processes.

### A. Tensorial representation

Tensors are elegant mathematical objects that generalize the concepts of scalars, vectors, and matrices. A tensorial representation provides a natural and concise framework for modeling and solving multidimensional problems and is widely used in different fields, from linear algebra to physics. In particular, general relativity is completely formulated under the tensorial notation. Here, we use the representation formerly presented in Ref. [29]. We also adopt the Einstein summation convention, in order to have more compact equations: If two indices are repeated, where one is a superscript and the other a subscript, then such an operation implies a summation. Aside from that, the result is a tensor whose rank lowers by 2. For instance, $A_{\beta }^{\alpha }A_{\alpha }^{\gamma }=\underset{\alpha }{\sum }A_{\beta }^{\alpha }A_{\alpha }^{\gamma }$. In our notation, we use Greek letters to indicate the components of a tensor. In addition, we use a tilde ($\overset{˜}{\cdot }$) to denote the components related to the layers, with dimension m, while the components without a tilde have dimension n and are related to the nodes.

A multilayer network is represented as the fourth-order adjacency tensor $M\in R^{n\times n\times m\times m}$, which can represent several relations between nodes [29],

$$M_{\beta \overset{˜}{\gamma }}^{\alpha \overset{˜}{\delta }}=\overset{m}{\underset{\overset{˜}{h},\overset{˜}{k}=1}{\sum }}C_{\beta }^{\alpha }(\overset{˜}{h}\overset{˜}{k})E_{\overset{˜}{\gamma }}^{\overset{˜}{\delta }}(\overset{˜}{h}\overset{˜}{k})\;=\overset{m}{\underset{\overset{˜}{h},\overset{˜}{k}=1}{\sum }}\overset{n}{\underset{i,j=1}{\sum }}w_{ij}(\overset{˜}{h}\overset{˜}{k})E_{\beta \overset{˜}{\gamma }}^{\alpha \overset{˜}{\delta }}(ij\overset{˜}{h}\overset{˜}{k}),$$ (1)

where $E_{\tilde{\delta }}^{\tilde{\gamma }}(\tilde{h}\tilde{k})\in R^{m\times m}$ and $E_{\beta \tilde{\gamma }}^{\alpha \tilde{\delta }}(ij\tilde{h}\tilde{k})\in R^{n\times n\times m\times m}$ indicate the tensor in its respective canonical basis. Observe that we can extract one layer by projecting the tensor $M_{\beta \tilde{\gamma }}^{\alpha \tilde{\delta }}$ to the canonical tensor $E_{\tilde{\delta }}^{\tilde{\gamma }}(\tilde{r}\tilde{r})$. Formally, from Ref. [29], we have

$$M_{\beta \tilde{\gamma }}^{\alpha \tilde{\delta }}E_{\tilde{\delta }}^{\tilde{\gamma }}(\tilde{r}\tilde{r})=C_{\beta }^{\alpha }(\tilde{r}\tilde{r})=A_{\beta }^{\alpha }(\tilde{r}),$$ (2)

where $\tilde{r}\in \{1,2,\ldots ,m\}$ is the selected layer and $A_{\beta }^{\alpha }(\tilde{r})$ is the adjacency matrix (rank-2 tensor). Moreover, aiming at having more compact and clear equations, we define the all-one tensors $u_{\alpha }\in R^{n}$ and $U^{\beta \tilde{\delta }}\in R^{n\times m}$. Here, we restrict our analysis to multilayer networks with a diagonal coupling [27]. In other words, each node can have at most one counterpart on the other layers. In addition, for simplicity, we focus on unweighted and undirected connected networks, in which there is a path from each node to all other nodes. For complementary information about the tensorial representation, its projections, and the generalization of the eigenvalue problem, see Appendix A.

### B. Susceptible-infected-susceptible model

Despite its simplicity, the SIS and SIR models capture the main features of disease spreading [1]. In this section, we focus on the first-order approximation of the SIS model. Additionally, we present some aspects of the SIS exact formulation in Appendix B 1 and a brief analysis of the SIR model in Appendix C.

We model the SIS disease dynamics associating a Poisson process with each of the elementary dynamical transitions: intralayer and interlayer spreading and the recovery from the infected state. The first two processes are associated with the edges of the graph and are characterized by the parameters λ and η, respectively. The latter transition is modeled in the node, also via a Poisson process with parameter μ. Using the tensorial notation defined above, the equations describing the system dynamics read

$$\frac{dX_{\beta \tilde{\delta }}}{dt}=-\mu X_{\beta \tilde{\delta }}+\left(1-X_{\beta \tilde{\delta }}\right)\lambda R_{\beta \tilde{\delta }}^{\alpha \tilde{\gamma }}(\lambda ,\eta )X_{\alpha \tilde{\gamma }},$$ (3)

where the supra-contact tensor is defined as

$$R_{\beta \tilde{\delta }}^{\alpha \tilde{\gamma }}(\lambda ,\eta )=M_{\beta \tilde{\sigma }}^{\alpha \tilde{\eta }}E_{\tilde{\eta }}^{\tilde{\sigma }}(\tilde{\gamma }\tilde{\delta })\delta_{\tilde{\delta }}^{\tilde{\gamma }}+\frac{\eta }{\lambda }M_{\beta \tilde{\sigma }}^{\alpha \tilde{\eta }}E_{\tilde{\eta }}^{\tilde{\sigma }}(\tilde{\gamma }\tilde{\delta })(U_{\tilde{\delta }}^{\tilde{\gamma }}-\delta_{\tilde{\delta }}^{\tilde{\gamma }}),$$ (4)

which encodes the contacts. It has a similar role as the matrix R in Ref. [28]. Notice that we have implicitly assumed that the random variables $X_{\beta \tilde{\delta }}$ are independent. Formally, if the state variable (Bernoulli random variable) $S_{\beta \overset{˜}{\delta }}$ is such that $S_{\beta \overset{˜}{\delta }}=1$ when the node β on layer $\tilde{\delta }$ is a spreader and $S_{\beta \overset{˜}{\delta }}=0$ otherwise, then $P[S_{\beta \overset{˜}{\delta }}=1]=X_{\beta \overset{˜}{\delta }}$. In this way, the independence of random variables implies that $P[S_{\beta \overset{˜}{\delta }}=1,S_{\alpha \overset{˜}{\gamma }}=1]=P[S_{\beta \overset{˜}{\delta }}=1]P[S_{\alpha \overset{˜}{\gamma }}=1]=X_{\beta \overset{˜}{\delta }}X_{\alpha \overset{˜}{\gamma }}$. Cator and Van Mieghem [33] proved rigorously that the states of any two nodes in the SIS model are non-negatively correlated for all finite graphs. This result can be easily extended to our case since we are considering constant rates and Markovian processes. Because of the positive contribution of the infected nodes, we have $P[S_{\beta \overset{˜}{\delta }}=1|S_{\alpha \overset{˜}{\gamma }}=1]\geq P[S_{\beta \overset{˜}{\delta }}=1]$, implying that the model is always overestimated. A similar conclusion was also obtained in Ref. [13] for the monolayer case.

Naturally, the order parameter, also called the macrostate variable, is defined as the average of the individual probabilities, formally given by

$$\rho =\frac{1}{nm}X_{\beta \tilde{\delta }}U^{\beta \tilde{\delta }}.$$ (5)

Note that the steady state is not an absorbing state in the Markov sense since there is a set of possible states where the system remains trapped and there is a stochastic variation over time. In addition, note that there are many different configurations for which the fraction of infected nodes is the same. More formally, there is a set of states above the threshold, which have finite probability larger than zero, configuring a metastate. The only absorbing state of this set of equations is thus the disease-free state since when it is reached, the (micro and macro) dynamics stops.

Furthermore, one of the most important concepts of disease-spreading processes is the epidemic threshold: Before the threshold, the system is in a disease-free state. On the other hand, when increasing the spreading rate, it drives the population to an endemic state. In other words, there is a nonzero probability that the disease remains in the population, configuring the metastate described above. Analogously to the results for monolayer systems, we have a critical point given as

$${\left(\frac{\mu }{\lambda }\right)}_{c}=\Lambda_{1},$$ (6)

where $\Lambda_{1}$ is the largest eigenvalue of R. The complete derivation of the critical point is presented in Appendix B 2. Observe that the eigenstructure of the tensor R is the same as for the matrix R in Ref. [28] since it can be understood as a flattened version of the tensor $R_{\beta \tilde{\delta }}^{\alpha \tilde{\gamma }}(\lambda ,\eta )$. As argued in Ref. [29], the supra-adjacency matrix corresponds to a unique unfolding of the fourth-order tensor R yielding square matrices. Moreover, if $\eta M_{\nu \tilde{\delta }}^{\xi \tilde{\gamma }}E_{\xi }^{\nu }(\beta \beta )\ll \lambda M_{\beta \tilde{\gamma }}^{\alpha \tilde{\xi }}E_{\tilde{\xi }}^{\tilde{\gamma }}(\tilde{\delta }\tilde{\delta })$, the critical point is dominated by the individual-layer behavior and the epidemic threshold is approximated to that of a SIS model on monolayers, when considering the union of m disjoint networks. Consequently, the epidemic threshold is determined by the largest eigenvalue, considering all layers. The same conclusion was reached in Ref. [28] using perturbation theory on the supra-contact matrix.

Finally, the nodal probability on the steady state can be bounded by

$$1-\frac{1}{1+\frac{d_{\beta \tilde{\delta }}}{d^{\min}}\left[\left(\frac{\lambda }{\mu }\right)d^{\min}-1\right]}\leq X_{\beta \tilde{\delta }}^{\infty }\leq 1-\frac{1}{\left(\frac{\lambda }{\mu }\right)d_{\beta \tilde{\delta }}+1},$$ (7)

where $X_{\beta \tilde{\delta }}^{\infty }$ denotes the probability that node β in layer $\tilde{\delta }$ is in the steady-state regime, $d_{\beta \tilde{\delta }}=R_{\beta \tilde{\delta }}^{\alpha \tilde{\gamma }}(\lambda ,\eta )U_{\alpha \tilde{\gamma }}$ [also defined in (B9)] and $d^{\min}=\min\{d_{\beta \tilde{\delta }}\}$. The derivations of such bounds are shown in detail in Appendix B 3. Interestingly, observe that the higher $d^{\min}$, the closer the lower and upper bounds. In the extreme case (λ/μ)→∞, the bounds approach each other and all nodes tend to be infected. Phenomenologically, the latter parameter configuration models the limiting case of a SI-like scenario, where μ=0. In such a dynamical process, all individuals are infected in the steady state.

## III. SPECTRAL ANALYSIS OF R(λ,η)

As observed in the previous section, the supra-adjacency tensor R(λ,η) plays a major role in the epidemic process. Consequently, a deeper analysis of the spectral properties of such objects can give us further insights about the whole process. First of all, the generalization of the eigenvector problem to the eigentensor is described in Appendix A 2, allowing us to use some well-established linear algebra tools. Additionally, in this section, we generalize the spectral results of interlacing, obtained in Refs. [30,34], to the tensorial description adopted here. We also make use of the inverse participation ratio [IPR(Λ)] as a measurement of eigenvalue localization [31]. As a convention, we assume that the eigenvalues are ordered as $\Lambda_{1}\geq \Lambda_{2}\geq \ldots \Lambda_{nm}$ and the individual-layer eigenvalues are denoted as $\Lambda_{i}^{l}$.

### A. Interlacing properties

**FIG. 1.**
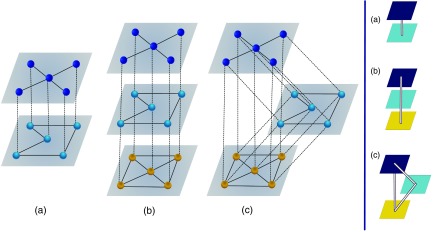
Schematic illustration of the three multilayer network cases considered as examples. The left panels represent the original networks, which give rise to three distinct configurations for the networks of layers (right panels). See the text for more details.

Invoking the unique mapping presented in Appendix A 2 and considering the results of Refs. [30,34], we can use the interlacing properties to relate the spectra of the multilayer network with the spectra of the network of layers. First, we define the normalized network of layers in terms of the supra-contact tensor as

$$\Phi_{\tilde{\delta }}^{\tilde{\gamma }}(\lambda ,\eta )=\frac{1}{n}R_{\beta \tilde{\delta }}^{\alpha \tilde{\gamma }}(\lambda ,\eta )U_{\alpha }^{\beta },$$ (8)

where we are implicitly assuming a multilayer network in which the layers have the same number of nodes and a dependency on the spreading rates (the demonstration that such a tensor is an unfolding of the matrix exposed in Ref. [30] is shown in Appendix A 3). Additionally, let us denote by $\mu_{1}\geq \mu_{2}\geq \ldots \geq \mu_{m}$ the ordered eigenvalues of $\Phi_{\tilde{\delta }}^{\tilde{\gamma }}(\lambda ,\eta )$. Following Ref. [30], the interlacing properties imply

$$\Lambda_{nm-m+j}\leq \mu_{j}\leq \Lambda_{j},$$ (9)

for j=m,…,1. As examples, Table I shows the spectrum of three simple networks of layers that can be computed analytically: a line with two and three nodes and a triangle. Figure 1 shows a schematic illustration of those three multilayer networks.

**TABLE I.** Structure and spectra of the normalized network of layers $\Phi_{\tilde{\delta }}^{\tilde{\gamma }}(\lambda ,\eta )$. The eigenvalues assumes that the average degree of each layer, $\left\langle k^{l}\right\rangle$, is the same, i.e., $\left\langle k^{l}\rangle =\langle k\right\rangle$, ∀  l.

Network	$\Phi_{\tilde{\delta }}^{\tilde{\gamma }}(\lambda ,\eta )$	Eigenvalues
Line with two nodes	$\left[\begin{array}{cc} \left\langle k^{l=1}\right\rangle & \eta /\lambda \\ \eta /\lambda & \left\langle k^{l=2}\right\rangle \end{array}\right]$	⟨k⟩-(η/λ)⟨k⟩+(η/λ)
Line with three nodes	$\left[\begin{array}{ccc} \left\langle k^{l=1}\right\rangle & \eta /\lambda & 0\\ \eta /\lambda & \left\langle k^{l=2}\right\rangle & \eta /\lambda \\ 0 & \eta /\lambda & \left\langle k^{l=3}\right\rangle \end{array}\right]$	⟨k⟩$\left\langle k\rangle -\sqrt{2}(\eta /\lambda \right)$$\left\langle k\rangle +\sqrt{2}(\eta /\lambda \right)$
Multiplex	$\left[\begin{array}{ccc} \left\langle k^{l=1}\right\rangle & \eta /\lambda & \eta /\lambda \\ \eta /\lambda & \left\langle k^{l=2}\right\rangle & \eta /\lambda \\ \eta /\lambda & \eta /\lambda & \left\langle k^{l=3}\right\rangle \end{array}\right]$	⟨k⟩-(η/λ)⟨k⟩-(η/λ)⟨k⟩+2(η/λ)

Furthermore, using similar arguments, we can also obtain results for the normalized projection, formally given as

$$P_{\beta }^{\alpha }=\frac{1}{m}R_{\beta \tilde{\delta }}^{\alpha \tilde{\gamma }}(\lambda ,\eta )U_{\tilde{\gamma }}^{\tilde{\delta }},$$ (10)

whose ordered eigenvalues, denoted by $\nu_{1}\geq \nu_{2}\geq \ldots \geq \nu_{m}$, also interlace with the supra-contact tensor satisfying

$$\Lambda_{nm-n+j}\leq \nu_{j}\leq \Lambda_{j},$$ (11)

for j=n,…,1. Finally, the adjacency tensor of an extracted layer also interlaces, yielding

$$\Lambda_{nm-n+j}\leq \Lambda_{j}^{l}\leq \Lambda_{j},$$ (12)

for j=n,…,1. These results show that the eigenvalue of the multilayer adjacency tensor is always larger than or equal to all of the eigenvalues of the individual isolated layers, as well as the network of layers.

The interlacing properties presented here imply some constraints on the epidemic threshold. As in Ref. [30], let $\Lambda_{i}(M)$ be the ith eigenvalue of the tensor M and consider that the set of eigenvalues is ordered as before. Moreover, for simplicity, we suppress the argument when referring to the supra-contact matrix. First, assuming a fixed ratio of spreading rates, we observe that the eigenvalue of the multilayer follows,

$${\left(\frac{\lambda }{\mu }\right)}_{c}^{\tilde{r}}=\frac{1}{\Lambda_{1}(A_{\beta }^{\alpha }(\tilde{r}))}\geq \frac{1}{\Lambda_{1}},\;\forall \;\;\tilde{r}\in 1,2,\ldots ,m,$$ (13)

where ${\left(\lambda /\mu \right)}_{c}^{\overset{˜}{r}}$ is the critical point for the single layer $\tilde{r}$ and

$${\left(\frac{\lambda }{\mu }\right)}_{c}^{\Phi }=\frac{1}{\Lambda_{1}(\Phi_{\tilde{\delta }}^{\tilde{\gamma }})}\geq \frac{1}{\Lambda_{1}},$$ (14)

where ${\left(\lambda /\mu \right)}_{c}^{\Phi }$ denotes the critical point of the network of layers. Finally, considering the projection, we get

$${\left(\frac{\lambda }{\mu }\right)}_{c}^{P}=\frac{1}{\Lambda_{1}(P_{\beta }^{\alpha })}\geq \frac{1}{\Lambda_{1}},$$ (15)

where ${\left(\lambda /\mu \right)}_{c}^{P}$ is the critical point of the normalized projection. Thus, the spreading process on the whole system is at least as efficient as it is on the layers and on the network of layers. Note that efficiency is understood here in terms of the position of the critical point and not regarding the fraction of infected individuals in the steady state.

### B. Localization and spreading of diseases

Next, we investigate the behavior of the system near the phase transition and whether the phenomenon of disease localization shows up. These two issues were explored for monoplex networks in Refs. [10] and [31], respectively, but have not been addressed for the case of multilayer systems. The nodal probabilities can be written as a linear combination of the eigenbasis of R as

$$X_{\beta \tilde{\delta }}=\sum_{\Lambda }c(\Lambda )f_{\beta \tilde{\delta }}(\Lambda ),$$ (16)

where c(Λ) are the projections of $X_{\beta \tilde{\delta }}$ on the eigentensors f. Similarly to Ref. [31], substituting such an expression in the middle term of Eq. (B7), we obtain

$$c(\Lambda )=\sum_{\alpha \tilde{\gamma }}\frac{\lambda \sum_{\Lambda^{\prime }}c(\Lambda^{\prime })\Lambda^{\prime }f_{\alpha \tilde{\gamma }}(\Lambda^{\prime })f_{\alpha \tilde{\gamma }}(\Lambda )}{\lambda \sum_{\Lambda^{\prime }}c(\Lambda^{\prime })\Lambda^{\prime }f_{\alpha \tilde{\gamma }}(\Lambda^{\prime })+\mu }.$$ (17)

Considering only the contributions of the first eigenvalue and eigentensor, for $\lambda \geq \lambda_{c}$, the first-order approximation of the macrostate parameter is $\rho \approx \alpha_{1}\tau$, where $\tau =[(\lambda /\mu )/\Lambda_{1}-1]$, which yields

$$\alpha_{1}=\frac{f_{\beta \tilde{\delta }}(\Lambda_{1})U^{\beta \tilde{\delta }}}{nm[f_{\beta \tilde{\delta }}(\Lambda_{1}){]}^{3}U^{\beta \tilde{\delta }}}.$$ (18)

Such an expression is exact if there is a gap between the first two eigenvalues [10,31]. Furthermore, considering two eigentensors, we have $\rho \approx \alpha_{1}\tau +\alpha_{2}\tau^{2}$. In addition, following a similar approach as in Ref. [31], we can use the inverse participation ratio:

$$\mathrm{IPR}(\Lambda )\equiv {\left(f_{\beta \tilde{\delta }}(\Lambda )\right)}^{4}U^{\beta \tilde{\delta }}.$$ (19)

In the limit of nm→∞, if the IPR(Λ) is of order O(1), the eigentensor is localized and the components of $f_{\beta \tilde{\delta }}(\Lambda )$ are of order O(1) only for a few nodes. On the other hand, if IPR(Λ)→0, then this state is delocalized and the components of $f_{\beta \overset{˜}{\delta }}(\Lambda )∼O\left(1/\sqrt{nm}\right)$. Additionally, another possible scenario, completely different from the traditional single-layer one, is possible if we consider layerwise localization, i.e., localization on layers instead of on a fraction of nodes. In such a case, the IPR(Λ) will be of order O(1/n) in the localized phase, whereas it will be of order O(1/nm) in the delocalized phase. This is because, in the layerwise localized phase, the components of the eigentensor are of order $O(1/\sqrt{n})$ for all the nodes in the dominant layer and of order zero for nodes in other layers. Thus, one easily realizes that the correct finite-size scaling to take in order to characterize such a transition is m→∞; i.e., the number of layers goes to infinity while the number of nodes per layer remains constant. In fact, in this limit, IPR(Λ) will vanish on one side of the transition point while remaining finite on the other side. In this way, we can also observe localized states in the case in which there is no possibility for localization in each of the layers if they were isolated.

## IV. MONTE CARLO SIMULATIONS

We next compare the analytical results with Monte Carlo simulations of the spreading process. The method proposed in Refs. [11,35] is adapted here to the case of multilayer networks. At each time step, the time is incremented by $\Delta t=[1/(\mu N_{i}+\lambda N_{k}+\eta N_{m})]$, where $N_{i}$ is the number of infected nodes, and $N_{k}$ and $N_{m}$ are the number of intralayer and interlayer edges emanating from them, respectively. With probability $\mu N_{i}/(\mu N_{i}+\lambda N_{k}+\eta N_{m})$, one randomly chosen infected individual becomes susceptible. On the other hand, with probability $\lambda N_{k}/(\mu N_{i}+\lambda N_{k}+\eta N_{m})$, one infected individual, chosen with a probability proportional to its intralayer degree, spreads the disease to an incident edge chosen uniformly at random. Finally, with probability $\eta N_{m}/(\mu N_{i}+\lambda N_{k}+\eta N_{m})$, one infected individual, chosen with a probability proportional to its interlayer degree, propagates the disease to an edge chosen uniformly. If an edge between two infected individuals is selected during the spreading, nothing happens; only time is incremented. The process is iterated following this set of rules, simulating the continuous process described by the SIS scenario.

The quasistationary state (QS) method [11,35] restricts the dynamics to nonabsorbing states. Every time the process tries to visit an absorbing state, it is substituted by an active configuration previously visited and is stored on a list with M configurations, constantly updated. With a probability $p_{r}$, a random configuration on such a list is replaced by the actual configuration. In order to extract meaningful statistics from the quasistatic distribution, denoted by $\bar{P}(n^{I})$, where $n^{I}$ is the number of infected individuals, the system must be on the stationary state and a large number of samples must be extracted. In this way, we let the simulations run during a relaxation time $t_{r}$ and extract the distribution $\bar{P}(n^{I})$ during a sampling time $t_{a}$. The threshold can be estimated using the modified susceptibility [11], given by

$$\chi =\frac{\langle (n^{I}{)}^{2}\rangle -\langle n^{I}\rangle^{2}}{\left\langle n^{I}\right\rangle }=nm\left(\frac{\langle (\rho^{\mathrm{QS}}{)}^{2}\rangle -\langle \rho^{\mathrm{QS}}\rangle^{2}}{\left\langle \rho^{\mathrm{QS}}\right\rangle }\right),$$ (20)

where $\rho^{\mathrm{QS}}$ is the quasistationary distribution $\bar{P}(n^{I})$. As argued in Refs. [11,35], the susceptibility presents a peak at the phase transition on finite systems. Such a measure is the coefficient of variation of the temporal distribution of states over time on the steady state. Note that the magnitude of the susceptibility χ is not of primary interest to us, but rather the position of its maximum value with respect to λ/μ is of interest since it will coincide with the critical threshold for sufficiently large systems.

In addition, after obtaining the curves of χ×λ by the QS method, we also apply a moving average filter in order to get rid of the noise. Such an approach improves the visual quality of the plots and does not interfere with the results since the order of magnitude of the noise is smaller than those of the peaks corresponding to the transition points.

The parameters used in the QS method are $p_{r}=0.01$, $t_{a}$ varies from $10^{5}$ to $10^{6}$, and $t_{r}$ varies from $10^{5}$ to $3\times 10^{6}$ in order to obtain a smoother curve. The QS method demands a large sample size since it is estimating the variance of a distribution. Moreover, we construct the χ×λ curves in steps of $\Delta \lambda =10^{-3}$, and the moving average window has five points.

## V. TWO-LAYER MULTIPLEX SYSTEMS

In this section, we numerically study two-layer multiplex systems. First, we focus on the phase diagram of the spreading process as a function of the interlayer and intralayer spreading rates for both SIS and SIR scenarios. Next, we analyze the spectral properties of such systems, comparing with results of Sec. III. Finally, we perform Monte Carlo simulations that show the existence of multiple susceptibility peaks on multiplex networks. The latter results are analyzed in terms of the spectral properties of R(λ,η).

### A. Numerical solution

Results shown in this section are the numerical solutions of the ODE systems (3) (SIS) and (C1) (SIR) using a Runge-Kutta (4,5) algorithm [36]. We consider a two-layer multiplex network (m=2), where each layer has $n=10^{4}$ nodes. In order to build a multiplex network where the epidemic thresholds associated with the individual layers are well separated, we must guarantee that $\Lambda_{1}^{l}\gg \Lambda_{2}^{l}$. Therefore, we chose the degree distribution of the first layer to be $P(k)∼k^{-2.5}$, whereas that of the second layer is $P(k)∼k^{-4.5}$. Both layers are created using the uncorrelated configuration model [37]. Moreover, we consider a multilayer network in which every node has its counterpart on the other layer. This pairing of nodes of different layers is made randomly. Each result is the solution considering one single (and fixed) multiplex network.

**FIG. 2.**
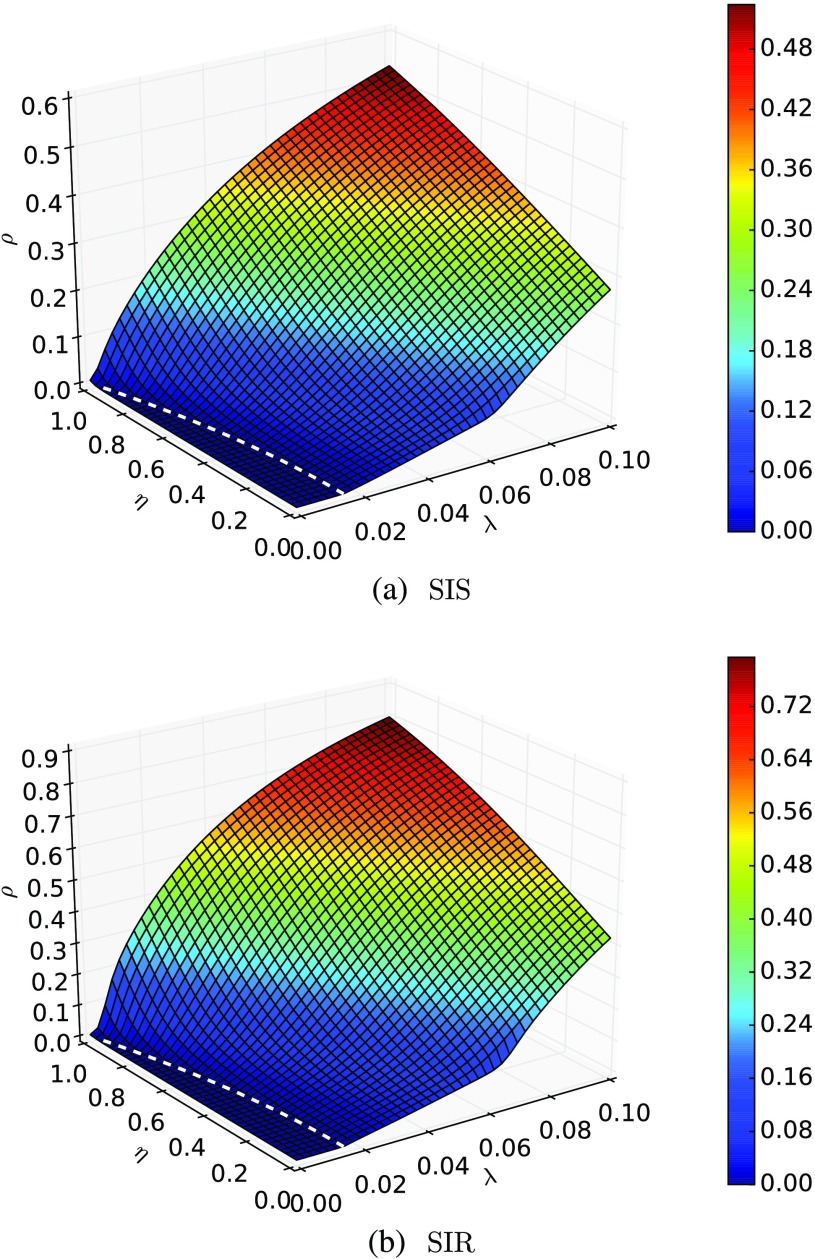
Phase diagrams over a two-layer multiplex system, where each layer is a scale-free network with $n=10^{4}$ nodes, for a fixed value of μ=1. (a) Density of spreaders as a function of the parameters η and λ. (b) Density of recovered individuals as a function of the parameters η and λ. Colors represent the fraction of spreaders, and the white line is the threshold calculated using Eq. (6).

Figure 2 shows the phase diagram considering the average fraction of spreaders for the SIS dynamics (or recovered for the SIR dynamics) as the macrostate variable as a function of the spreading parameters λ and η for a given recovering rate μ=1. The dashed white line denotes the epidemic threshold obtained from Eq. (6). In panel (a), we show the SIS scenario, while panel (b) corresponds to the SIR model. In both cases, it is possible to observe two changes in the system’s behavior. The first change is in the epidemic threshold, while the second is near the epidemic threshold of the second layer. In addition, we note the agreement between the theoretical epidemic thresholds and the numerical results. Furthermore, the higher η, the lower the epidemic threshold, which is a consequence of the eigentensor problem. Also note that ρ increases for a fixed λ as η increases, even for λ∼0, which means that in such extreme cases, the disease spreads mainly in the interlayer edges.

**FIG. 3.**
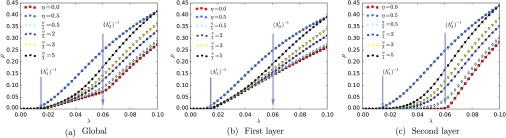
Individual layer behavior over a two-layer multiplex system. Each layer has $n=10^{4}$ for a fixed value of μ=1. The results considering both layers are shown in (a), while the dynamics in the individual layers are shown in (b) [$P(k)∼k^{-2.5}$] and (c) [$P(k)∼k^{-4.5}$]. The arrows indicate the leading eigenvalues of the layers.

Figure 3 shows the phase diagram for μ=1 and different values of the parameter η for the SIS dynamics. For η=0, we have no interlayer spreading, while for η=0.5, we have a fixed spreading rate, independent of the intralayer rates. In addition, we also evaluated cases where the ratio η/λ is constant. In Fig. 3(a), we show the global behavior of the system, which is an average of the individual behavior of the layers, represented in panels (b) and (c), since both layers have the same number of nodes. Furthermore, we also observe that the two individual networks show different behaviors near the epidemic threshold [10]. The first layer [Fig. 3(b)] has a lower epidemic threshold than the second. However, ρ grows (as a function of λ) slower in the first layer than in the second. This feature can be observed clearly in Figs. 3(b) and 3(c), where we show results for η=0, that is, when there is no spreading between the layers.

Considering the discrete system, Cozzo *et al.* [28] verified the shifting in the dominated layer (the largest amongst all individual eigenvalues) as the ratio η/λ increases. Here, we observe the same effect, as can be seen in Fig. 3(c). Additionally, we can also note another global change approximately beyond $\lambda >(\Lambda_{2}^{l}{)}^{-1}$. Our findings suggest the possibility of multiple phase transitions due to the multiplex structure of the network. It is noteworthy that, in spite of the similarities between our continuous model and the discrete model [28], both represent slightly different processes. In the continuous case, two events cannot happen at the same time. On the other hand, in the discrete model, every node contacts its neighbors in one discrete time step. Despite these differences, the results show that both the continuous and discrete formulations are phenomenologically similar.

### B. Spectral analysis

Since the epidemic process is described through the supra-adjacency tensor R(λ,η), its spectral properties give us some insights into the whole process, especially the critical properties of the systems under analysis. In this section, we focus on the spectral analysis of such a tensor as a function of the ratio η/λ, considering a two-layer multiplex network with two different layers; i.e., there is a distance between the leading eigenvalues of each layer. Some important aspects of the spectral properties are discussed in Appendix D, where we present an analytical approach to the problem of eigenvalue crossings (see Appendix D 1 a). We focus on two special cases in increasing order of complexity: (i) the identical case, presented in Appendix D 1 b, where both layers are exactly the same (i.e., there is a high correlation between the degree in each layer), and (ii) the nonidentical case, discussed in Appendix D 1 c, where both layers have the same degree distribution but different configurations.

In this section, we focus on the case of two different layer structures, with spaced leading eigenvalues. We consider a multiplex network made up of two scale-free networks with γ≈2.2 and γ≈2.8. Both layers have ⟨k⟩≈8 and $n=10^{3}$ nodes on each layer, and the leading eigenvalues are $\Lambda_{1}^{1}=42.64$ for the first and $\Lambda_{1}^{2}=21.29$ for the second.

**FIG. 4.**
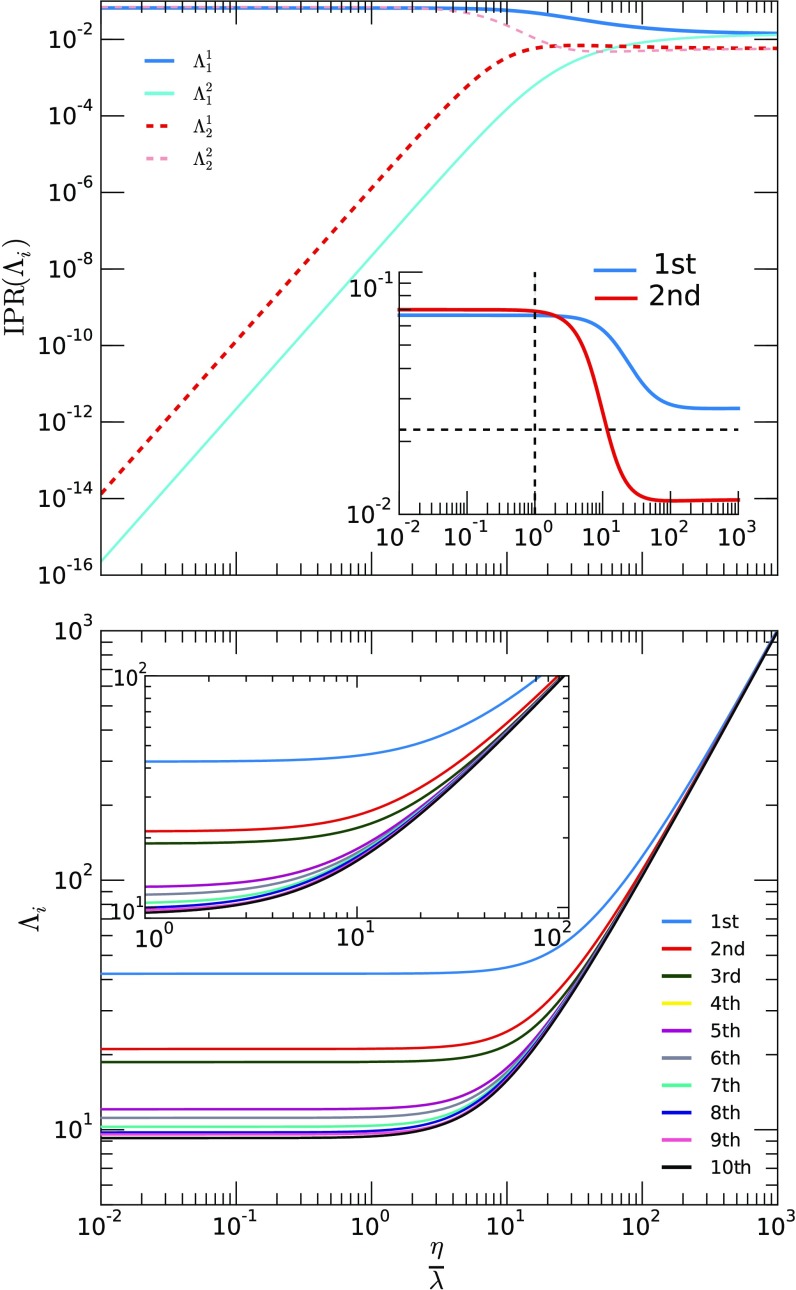
Spectral properties of the tensor R(λ,η) as a function of the ratio η/λ for a multiplex with two layers, the first with γ≈2.2 and the second with γ≈2.8. Both have ⟨k⟩≈8. In the top panel, we present the inverse participation ratio of the two larger eigenvalues and the individual-layer contributions, while in the bottom panel, we show the leading eigenvalues. Every curve is composed of $10^{3}$ log spaced points, in order to have enough resolution.

Figure 4 shows the spectral properties of the tensor R(λ,η) as a function of the ratio η/λ. In contrast to the identical layers (see Appendix D 1 b) and the case of statistically equivalent layers (Appendix D 1 c, Figs. 10 and 11), where some eigenvalues increase while others decrease, all the observed eigenvalues always increase in this case. Moreover, we do not observe any crossing or near-crossing behavior. Regarding IPR(Λ), the same pattern as for the similar case is found: For small values of η/λ and considering the first eigenvalue, the system appears localized in the first layer and delocalized in the second, while for $\mathrm{IPR}(\Lambda_{2})$, it is the contrary. For larger values of $\frac{\eta }{\lambda }$, both layers contribute equally to the IPR(Λ). Furthermore, the main difference we observe for the current setup with respect to the two similar networks (see Fig. 11 in Appendix D) is that now no drastic change in the inverse participation ratio is found, as expected, since there is no near crossing.

From Fig. 4, we can also extract an important numerical result regarding perturbation theory. We observed that in our case, considering a two spaced individual-layer eigenvalues problem, the leading eigenvalue can be approximated by the largest leading eigenvalue of the individual layers for (η/λ)≲1; such an approximation becomes less accurate as η/λ increases, but it can be acceptable up to (η/λ)≲10, within a certain error. Apart from that, note that both eigenvalues tend to increase, while their difference tends to decrease.

**FIG. 5.**
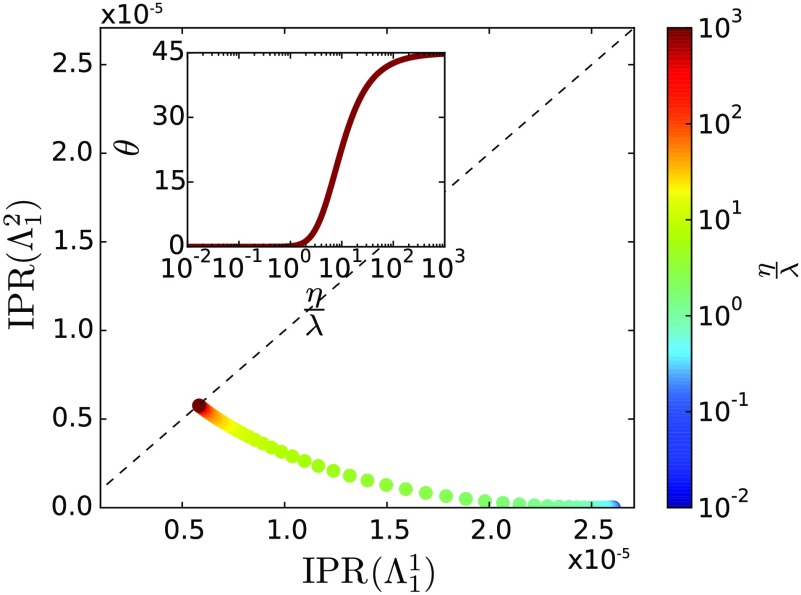
Diagram of the contribution of each layer to the IPR(Λ) for different values of the spreading ratio η/λ. The dashed line represents the case where both layers have the same contribution, i.e., a line with slope one. In the inset, we show the angle θ between the vector composed of the contributions of each layer to the IPR(Λ), $v={\left[\mathrm{IPR}(\Lambda_{1}^{1}),\mathrm{IPR}(\Lambda_{1}^{2})\right]}^{T}$, and the x axis. The multiplex network used here is composed of two Erdös-Rényi networks, both with $n=5\times 10^{4}$—the first layer is ⟨k⟩=16 [$(\Lambda_{1}^{1}{)}^{-1}\approx \;0.0625$], while the second is ⟨k⟩=12 [$(\Lambda_{1}^{2}{)}^{-1}\approx 0.0833$].

Furthermore, analyzing the eigenfunction properties, Fig. 5 shows the contribution of each layer to the IPR(Λ), considering different values of η/λ. Results correspond to a multiplex network composed of two Erdös-Rényi networks, both with $n=5\times 10^{4}$—the first layer with ⟨k⟩=16 and the second with ⟨k⟩=12. Observe that for lower values of η/λ, the main contribution comes from one layer, configuring a layerwise localized state and consequently placed on one axis (the x axis) of Fig. 5. Then, when the ratio η/λ increases, there is a transition to a delocalized state. This corresponds to an increase of the inverse participation ratio of the second layer; however, this increase is at the expense of decreasing the value of the inverse participation ratio of the first layer. In other words, in the localized phase, only the entries of the eigenvector associated with the dominant layer are effectively populated, while the entries associated with the other layers are not. In the delocalized phase, all the entries are equally populated. The inset of the figure further evidences this transition: It represents the angle θ between the vector composed of the IPR contributions, $v={\left[\mathrm{IPR}(\Lambda_{1}^{1}),\mathrm{IPR}(\Lambda_{1}^{2})\right]}^{T}$, and the x axis, where a change from zero to 45 degrees is observed as the ratio η/λ is increased and the system goes from a localized to a delocalized state.

### C. Multiple susceptibility peaks

Mata and Ferreira showed that it is possible to have multiple susceptibility peaks on monoplex networks [35]. They studied the behavior of a SIS model on networks with γ>3. Here, we show that such phenomena also appear, in a natural way, in multilayer networks. Motivated by the findings reported in the previous sections, especially by the presence of a second change in the slope of ρ as observed in Figs. 2 and 3, we have performed extensive Monte Carlo simulations using the QS method, with the aim of determining, as accurately as possible, the points at which the transitions take place for a two-layer multiplex network. Here, we use the multiplex from Sec. V B since the leading eigenvalues of each layer are spaced. Note that our numerical simulations are performed in a fixed network since we follow the quenched formalism.

**FIG. 6.**
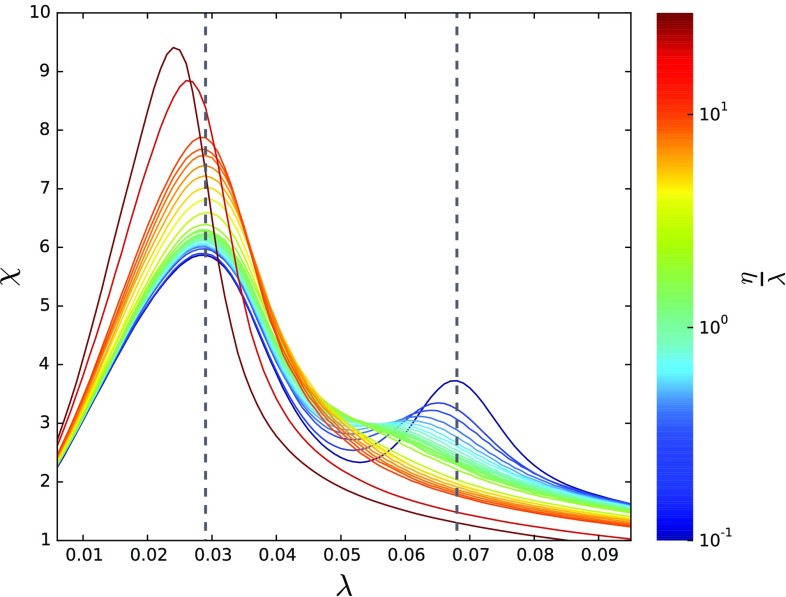
Susceptibility χ as a function of the spreading rate λ for different ratios of interlayer and intralayer spreading ratings, η/λ, for a fixed value of μ=1 over a two-layer multiplex system, where each layer has $n=10^{3}$—the first with γ≈2.2 and the second with γ≈2.8. Both have ⟨k⟩≈8. The simulated values are (η/λ)=0.1, 0.2, 0.3, 0.4, 0.5, 0.6, 0.7, 0.8, 0.9, 1.0, 1.1, 1.2, 1.3, 1.4, 1.5, 1.6, 2, 3, 4, 5, 6, 7, 8, 9, 10, 20, 30.

Figure 6 shows that for low values of the ratio η/λ, both networks are weakly coupled and the system exhibits two well-defined susceptibility peaks (vertical dotted lines). However, as this ratio increases, the peak signaling the presence of the second critical point decreases and eventually vanishes. In our simulations, we have observed that up to (η/λ)≈1, the second peak, although less defined, is still present. Beyond the latter point, only one peak remains. As η/λ further increases, the position of the critical point remains the same, and the peak is even more well defined. Interestingly enough, if the ratio η/λ continues to increase—in our case, beyond (η/λ)≳10—the critical point shifts to the left to values that are even smaller than the smallest critical point of the individual layers. It is worth highlighting that a similar qualitative behavior can be seen in the results shown in Fig. 2(a), where one can also observe a second change in the slope of ρ near the leading eigenvalue of the second layer. This change also vanishes as the intralayer spreading increases.

Since the tensor R(λ,η) plays a major role in the spreading process, our spectral results can help one understand the observed critical dynamics. In epidemiological terms—or, in general, for contagion processes—the localization of the disease in a certain layer means that most of the spreading is expected to take place on the nodes of that layer. Moreover, in addition to the localization in the layers, one can also have localization effects on specific nodes or groups of nodes, for instance.

In order to analytically explain this phenomenon, we evaluate IPR(Λ) for the two leading eigenvalues, as this measure indicates the localization of an eigenstate (see Sec. V B, results shown in Fig. 4). Comparing the susceptibility and IPR(Λ), we observe that $\mathrm{IPR}(\Lambda_{2})$ starts decaying for (η/λ)≈1 and crosses the value $1/\sqrt{nm}$, at which the associated eigenvector delocalizes, for (η/λ)≈10, comparing well with the point at which the second peak in the susceptibility decays and finally disappears. Moreover, $\mathrm{IPR}(\Lambda_{1})$ decays from 3≲(η/λ)≲10, which coincides with the range where the remaining maximum in the susceptibility reaches higher values and is better defined. More interestingly, note that $\mathrm{IPR}(\Lambda_{1})$ is mainly composed of the contributions of the first layer for a lower spreading ratio, suggesting that it is localized on such layer. Therefore, our results suggest that the IPR(Λ) is a proper measure to detect and predict the observed localization phenomena, and potentially for m localization transitions, as we will show in Sec. VI.

Regarding the definition of a critical point, it is important to highlight that the concept of phase transition only applies in the infinite size limit (the thermodynamic limit). However, in the literature of complex network dynamics, especially for epidemic spreading, it is common to use the terms “critical point” and “phase transition” in finite systems since we find a behavioral change at that point. More importantly, for scale-free networks, such a point vanishes in the thermodynamic limit. Following the usual convention in the complex network literature, the first susceptibility peak observed in all the experiments can be classified as a critical point of the phase transition. At such a point, the dynamics goes from a disease-free state to an endemic state. On the other hand, the second susceptibility peak cannot be classified this way since the process is already in an endemic state. Although it cannot be considered as a critical point, we have a transition from a layerwise localized state to a delocalized state. In other words, before the second susceptibility peak, most of the events take place in only one layer (the one with the largest individual eigenvalue), while after this point, both layers are active and spread the disease.

### D. Second susceptibility peak analysis: Erdös-Rényi layers

The second peak in the susceptibility curve suggests the existence of a second-order phase transition. However, from its existence alone, we cannot conclude this unequivocally since, although this point is related to the delocalization of the disease, the system is already in an endemic phase (upper critical regime, in physics jargon). Observe that if η∈O(1/n), in the thermodynamic limit, we would have a phase transition. However, such a configuration cannot be considered a multilayer network since both layers are (virtually) decoupled. Additionally, observe that we only analyzed layers without correlation. The presence of correlations can introduce different phenomena for discrete time (some were briefly explored in Ref. [32]), however, for discrete time.

In order to better understand the second peak of susceptibility, we analyze a two-layer multiplex network composed of two Erdös-Rényi networks, in which we can precisely control the mean degree and consequently the epidemic threshold by fixing the number of edges. Furthermore, for scale-free networks with a divergent second moment of its degree distribution, the epidemic threshold vanishes in the thermodynamic limit [1]. On the other hand, Erdös-Rényi networks always have a nonzero and finite critical point. Aside from that, since the nodes on such a network are statistically equivalent, the probabilities $X_{\beta \tilde{\delta }}$ are expected to be approximately the same. Henceforth, we assume that the first layer has a higher connectivity, that is, a lower epidemic threshold.

First, analyzing the layers individually for $(\lambda /\mu )>\;(\Lambda_{1}^{1}{)}^{-1}\geq \Lambda_{1}^{-1}$, the first layer is in its upper critical regime (endemic state), while the second layer is still in its subcritical regime (disease-free state). Then, for a coupling parameter, η>0, the probability of a node in the second layer being infected also increases. In fact, for Erdös-Rényi layers, it will always be larger than zero. Therefore, we can map this problem onto an ε-SIS model [38], where each node has a probability of experiencing a spontaneous infection. Note that such a model does not present an absorbing state. In this mapping, we are interested in the behavior of the second layer, and we consider that the self-infection ε is determined by the contribution of the first layer by means of the contacts between nodes in different layers, which are Poisson processes with parameter η. This would imply that we would not have a second-order phase transition. However, we have a transition from a layerwise localized system, in which only the first layer is active and able to sustain the disease for long times, to a delocalized system, where both layers are active.

In order to explore the time evolution of the system for a set of parameters near the second susceptibility peak, we run the continuous simulation 50 times and perform a moving average filter over a sampling of the original time series, resulting in $5\times 10^{4}$ points. This approach gives us an average curve over time. Note that for continuous simulations, the number of points can vary from one run to another. Both networks have $n=5\times 10^{4}$—the first with ⟨k⟩=16 [$(\Lambda_{1}^{1}{)}^{-1}\approx 0.0625$] and the second with ⟨k⟩=12 [$(\Lambda_{1}^{2}{)}^{-1}\approx 0.0833$].

**FIG. 7.**
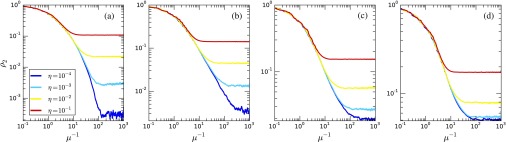
Time evolution of the fraction of infected nodes in the second layer for μ=1, different values of η ($10^{-4}$, $10^{-3}$, $10^{-2}$, and $10^{-1}$), and different values of the spreading rate: (a) λ=0.078, (b) λ=0.083, (c) λ=0.085, and (d) λ=0.088. The multiplex network used is composed of two Erdös-Rényi networks, both with $n=5\times 10^{4}$—the first layer with ⟨k⟩=16 [$(\Lambda_{1}^{1}{)}^{-1}\approx 0.0625$] and the second with ⟨k⟩=12 [$(\Lambda_{1}^{2}{)}^{-1}\approx 0.0833$].

Figure 7 shows the time evolution of a disease spreading in the second layer for different values of λ and η. The initial conditions for these experiments consider that the first layer has an initial probability of a node being infected equal to 0.01, while in the second, every node spreads. Note that we chose this initial condition for visual purposes since any initial condition would result in a similar steady-state regime. In this way, during the transient state, we observe a decay of the fraction of infected individuals; then, at the metastate that configures the steady state, we observe a stochastic variation centered on the average value. In addition, such fluctuations tend to increase near a “critical point.” We observe that for $(\Lambda_{1}^{1}{)}^{-1}>(\lambda ./\mu )>(\Lambda_{2}^{1}{)}^{-1}$ for $\eta =10^{-4}$, the incidence is very low, of order O(1/n); however, it is larger than zero. As we increase the value of λ, we drive the system to its active state, being able to sustain the disease and spreading it in the intra-edge contacts. In addition, by increasing η we are able to increase the incidence of the disease due to the intra-edge contacts. Near the critical point of the second layer, $(\lambda /\mu )=(\Lambda_{2}^{1}{)}^{-1}=0.833$, we can observe some features that are similar to a transition. From below, we observe that the lower the value of η, the longer it takes for the system to reach the steady state, similarly to what is expected in phase transitions. On the other hand, slightly above the critical point, the time it takes to get to the steady state decreases and the curves for $\eta =10^{-4}$ and $\eta =10^{-3}$ get closer. This suggests that the effects of intralayer spreading are the main source of spreading. Finally, for λ/μ sufficiently large, we observe the same behavior for all values of η; i.e., all of them are in an active state.

In addition to the analysis shown in this section, we also inspected, in detail, the steady state for different system sizes, showing that the fluctuations do not diverge and that the final fraction of infected individuals does not go to zero in the second layer. This analysis suggests that we do not have a second-order phase transition but that the dynamics changes from a layerwise localized to a delocalized phase. In this phenomenological scenario, the transition point is still of great importance for practical purposes—for instance, when studying immunization policies. These complementary results are shown in Appendix D 2.

## VI. THREE-LAYER INTERCONNECTED SYSTEMS: THE BARRIER EFFECT

Following the main ideas of the last sections, we explore the spreading dynamics in multilayer networks with more than two layers. Specifically, we have carried out numerical simulations for a three-layer system. We generate multiplex networks using three scale-free networks, with γ≈2.3, γ≈2.6, and γ≈2.9, with ⟨k⟩≈8 and $n=10^{3}$ nodes on each layer. Note that we consider three layers with spaced individual leading eigenvalues in order to investigate whether multiple susceptibility peaks are a generic phenomenon of multilayer systems. Note that we have two possible topologies for the network of layers: (i) a line graph and (ii) a triangle (which is a node-aligned multiplex). In its turn, the first can be arranged in three possible configurations by changing the central layer. In other words, we have four possible systems. In this section, we focus on two configurations, the multiplex case and the line (2.3+2.9+2.6). Both cases summarize the richness of dynamical processes in interconnected networks, presenting a new phenomenon, the barrier effect of an intermediate layer. We proceed by analyzing the spectral properties of this multilayer system in terms of the inverse participation ratio and the susceptibility. Regarding the other interconnected networks, we present those complementary results and analyses in Appendix E. Additionally, in Appendix E 1, we show that increasing η/λ also increases the role of the interlayer edges relative to the intralayer ones. Consequently, the structure of the network of layers imposes itself more strongly on the eigenvalues of the entire interconnected structure.

### A. Spectral analysis

**FIG. 8.**
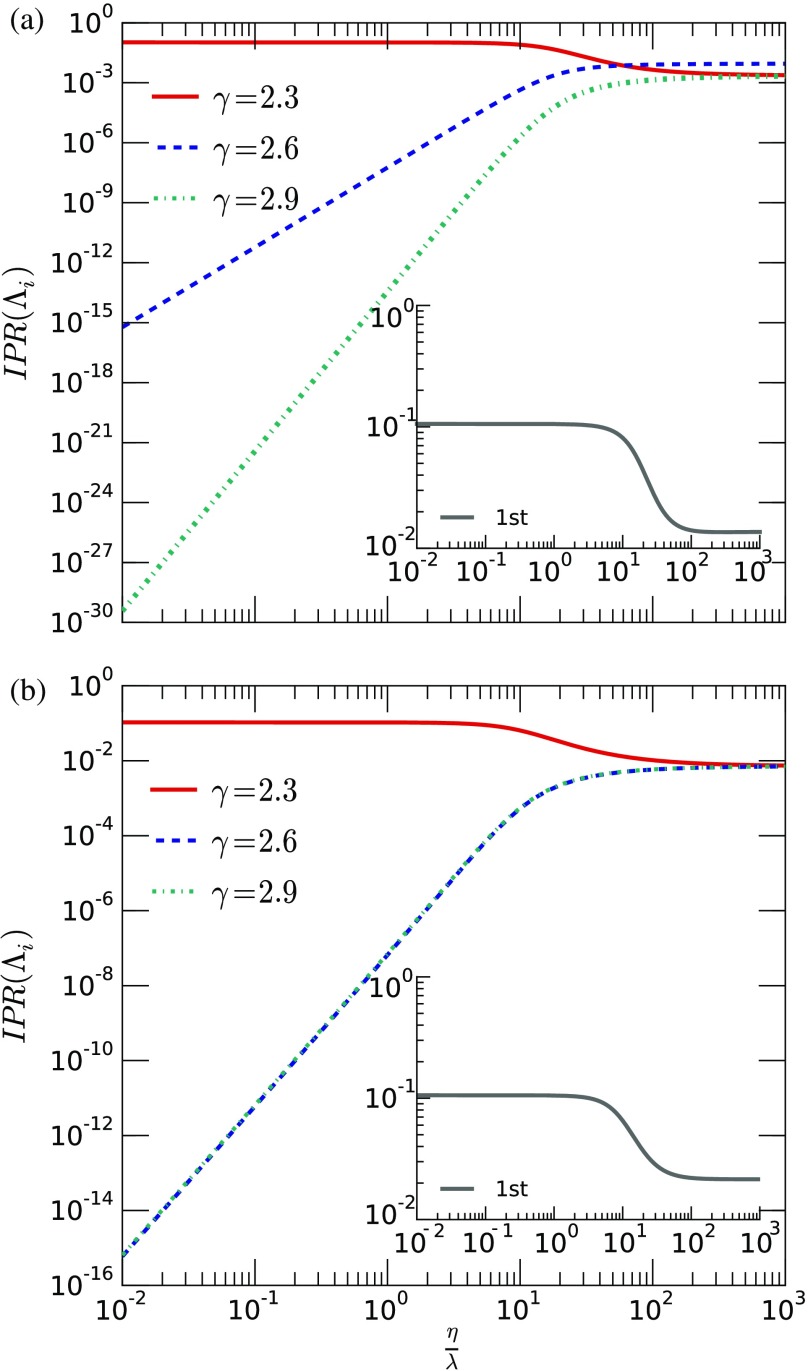
Spectral properties of the tensor R(λ,η) as a function of the ratio η/λ for a multiplex with two layers with the same degree distribution (different random realizations of the configuration model) and connected to its counterpart on the other layer. In the top panel, we present the IPR(Λ) of the two larger eigenvalues and the individual-layer contributions, while in the bottom panel, we show the leading eigenvalues. Every curve is composed by $10^{3}$ log spaced points, in order to have enough resolution. In panel (a), we show the line (2.3+2.9+2.6), while panel (b) is the multiplex case.

Figure 8 shows the $\mathrm{IPR}(\Lambda_{1})$ of tensor R. In the main panel, we present the individual contribution of each layer, while in the insets, we have the total $\mathrm{IPR}(\Lambda_{1})$. In the top panel, we have the line (2.3+2.9+2.6), whereas in the bottom panel, we have the multiplex network. In this section, we focus on the spectral comparison of two cases: (i) the lines (2.3+2.6+2.9) and (2.3+2.9+2.6) and (ii) the line (2.6+2.3+2.6) and the multiplex network. Additionally, the reader is referred to Appendix E 1, specifically to Fig. 17 for complementary results.

An interesting phenomenon can be observed by comparing the different configurations of the network of layers. The largest eigenvalue of the whole system, $\Lambda_{1}$, has its associated eigenvector localized in the dominant layer, that is, in the layer generated using γ=2.3. Regarding the line configuration, depending on the position of that layer in the whole system—i.e., central or peripheral layer—the contribution of the nondominant layers to the $\mathrm{IPR}(\Lambda_{1})$ varies. In particular, when the dominant layer corresponds to an extreme node of the network of layers, the contribution of the other two layers will be ordered according to the distance to the dominant one. Consequently, when the dominant layer is in the center of the network of layers, the contributions of the nondominant ones are comparable (see Fig. 17 in Appendix E 1 for complementary results).

Furthermore, for the first eigenvalue, which is usually enough to analyze the localization as a first-order approximation, we observe that the layer with the largest eigenvalue dominates the dynamics. In addition, note the similarities between the multiplex and the line configuration (2.6+2.3+2.6) (see also Fig. 17, Appendix E 1), where the nondominant layers behave similarly. This is because for small values of η/λ, the effect of the extra edge in the network of layers (closing the triangle) is of order $\eta^{2}$; thus, a similar behavior is observed for the two configurations. As η/λ grows, the symmetry in the node-aligned multiplex dominates the eigenvector structure, and the contributions of all layers are comparable. As we show next, the different contributions of the layers to the total $\mathrm{IPR}(\Lambda_{1})$ are at the root of the multiple susceptibility peaks observed.

### B. Multiple susceptibility peaks

**FIG. 9.**
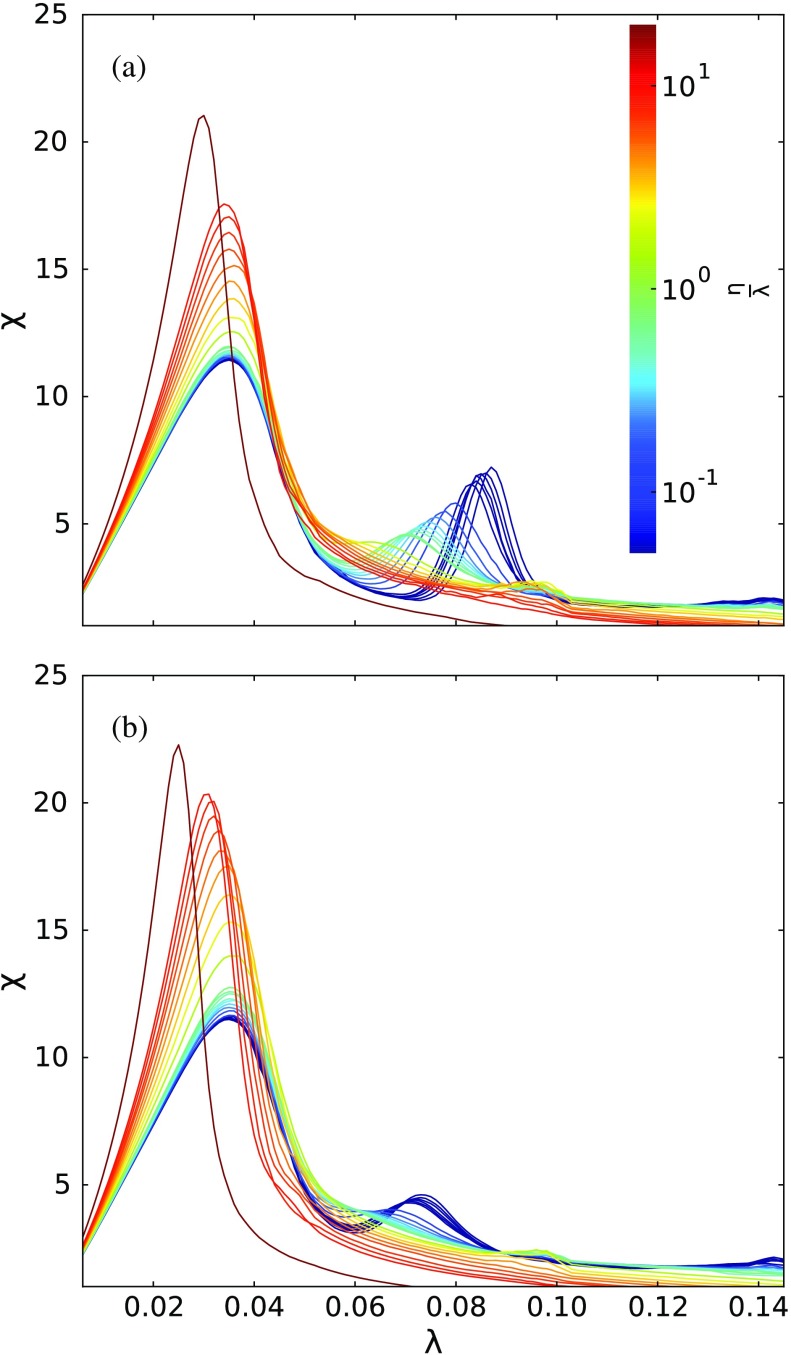
Susceptibility χ as a function of λ considering all three-layer configurations and many different ratios η/λ, which is represented by the color of the lines. The recovering rate is μ=1. The simulated values are (η/λ)=0.05, 0.06, 0.07, 0.08, 0.09, 0.1, 0.2, 0.3, 0.4, 0.5, 0.6, 0.7, 0.8, 0.9, 1.0, 2, 3, 4, 5, 6, 7, 8, 9, 10, 20. In panel (a), we show the line (2.3+2.9+2.6), while panel (b) is the multiplex case.

**FIG. 10.**
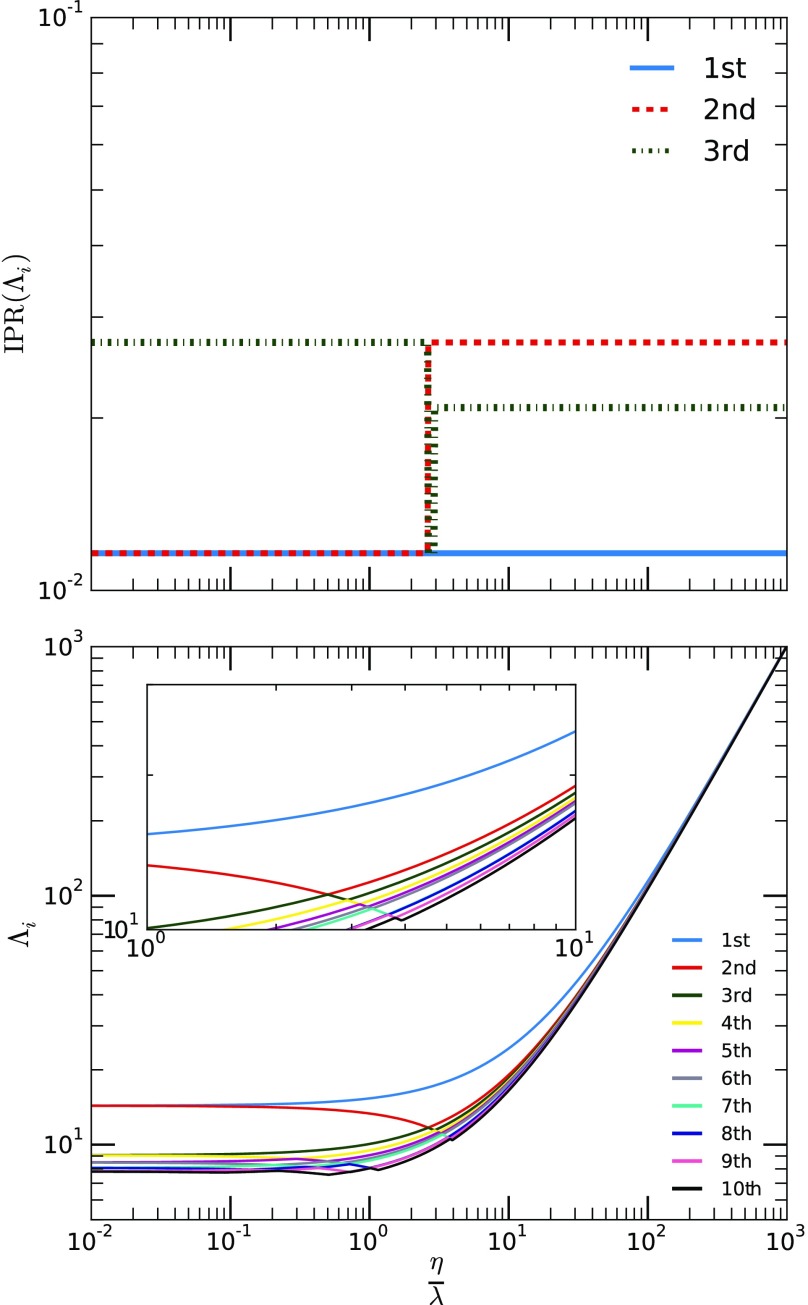
Spectral properties of the tensor R(λ,η) as a function of the ratio η/λ for a multiplex with two layers with the exact same degree distribution and connected to its counterpart on the other layer. In the top panel, we present the inverse participation ratio of the three larger eigenvalues, while in the bottom panel, we show the leading eigenvalues. Every curve is composed of $10^{3}$ log spaced points, in order to have enough resolution.

**FIG. 11.**
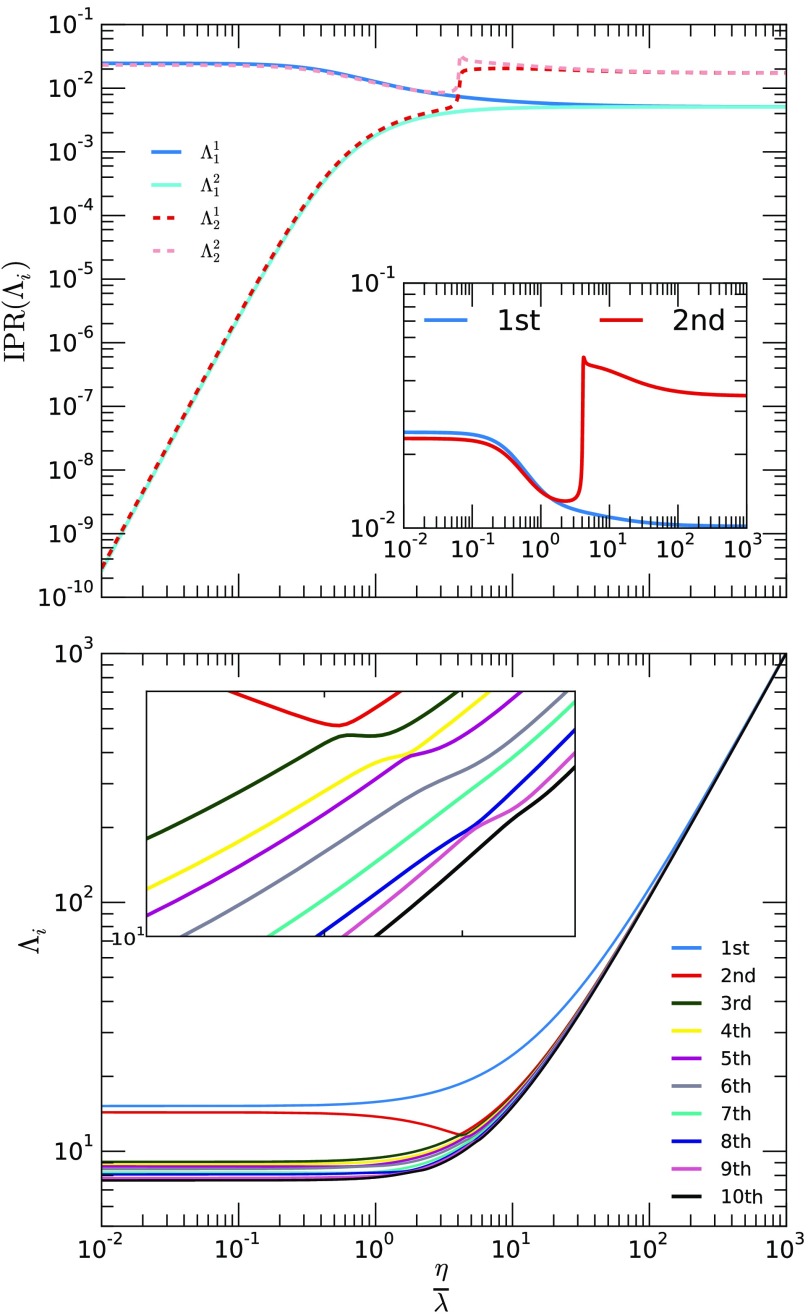
Spectral properties of the tensor R(λ,η) as a function of the ratio η/λ for a multiplex with two layers with the same degree distribution (different random realizations of the configuration model) and connected to its counterpart on the other layer. In the top panel, we present the inverse participation ratio of the two larger eigenvalues and the individual-layer contributions, while in the bottom panel, we show the leading eigenvalues. Every curve is composed of $10^{3}$ log spaced points, in order to have enough resolution.

**FIG. 17.**
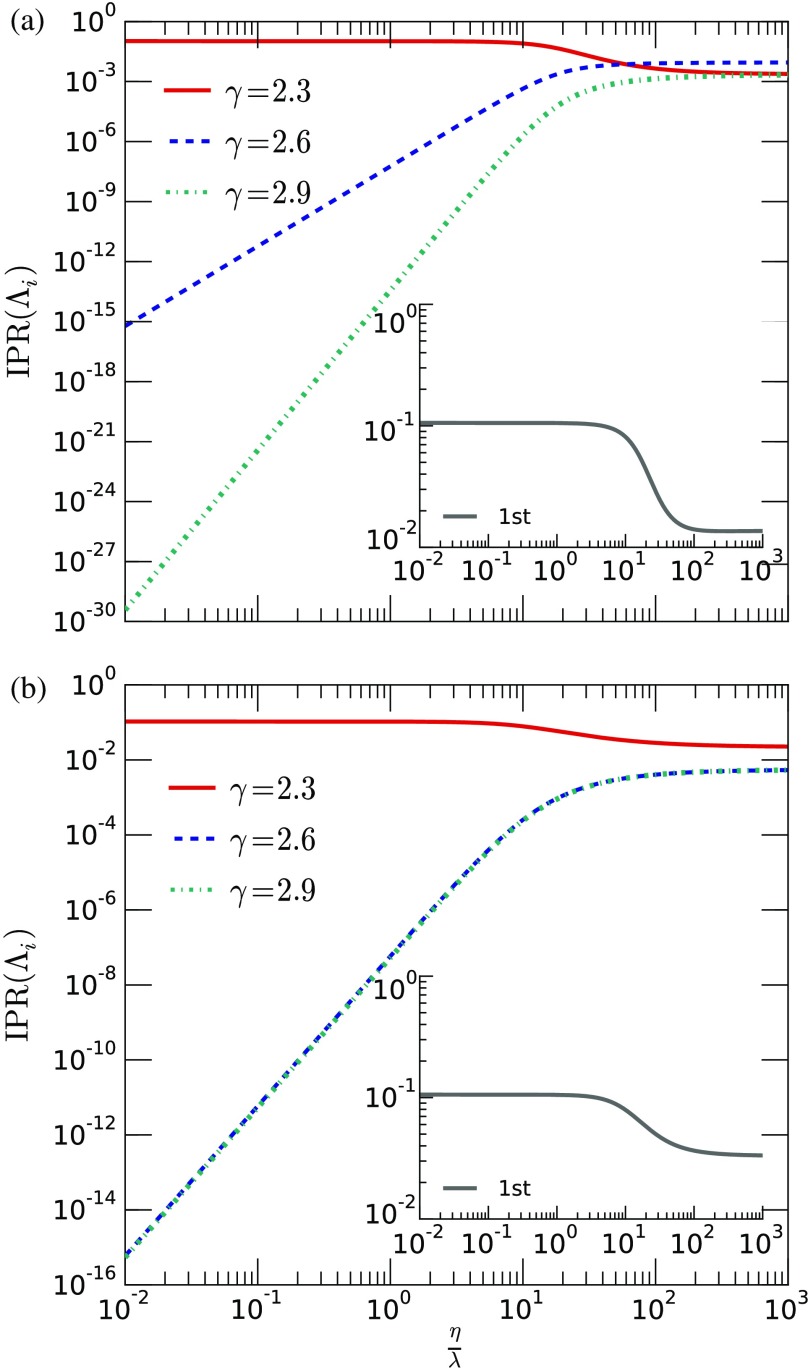
Spectral properties of the tensor R(λ,η) as a function of the ratio η/λ for a multiplex with two layers with the same degree distribution (different random realizations of the configuration model) and connected to its counterpart on the other layer. In the top panel, we present the inverse participation ratio of the two larger eigenvalues and the individual-layer contributions, while in the bottom panel, we show the leading eigenvalues. Every curve is composed of $10^{3}$ log spaced points, in order to have enough resolution.

Figure 9 shows the susceptibility as a function of λ for different ratios of η/λ. We observe three well-defined peaks on such curves when the ratio η/λ is small. In addition, similar to the two-layer case, such peaks tend to become less defined and vanish as the ratio η/λ increases. The third peak is less defined than the others because the average number of infected nodes is larger in this case. Consequently, the susceptibility tends to be lower since it measures the variance in relation to the average. Such an observation suggests that it could be harder to observe peaks for nondominating layers that have an individual critical point too far from the dominating layer.

**FIG. 18.**
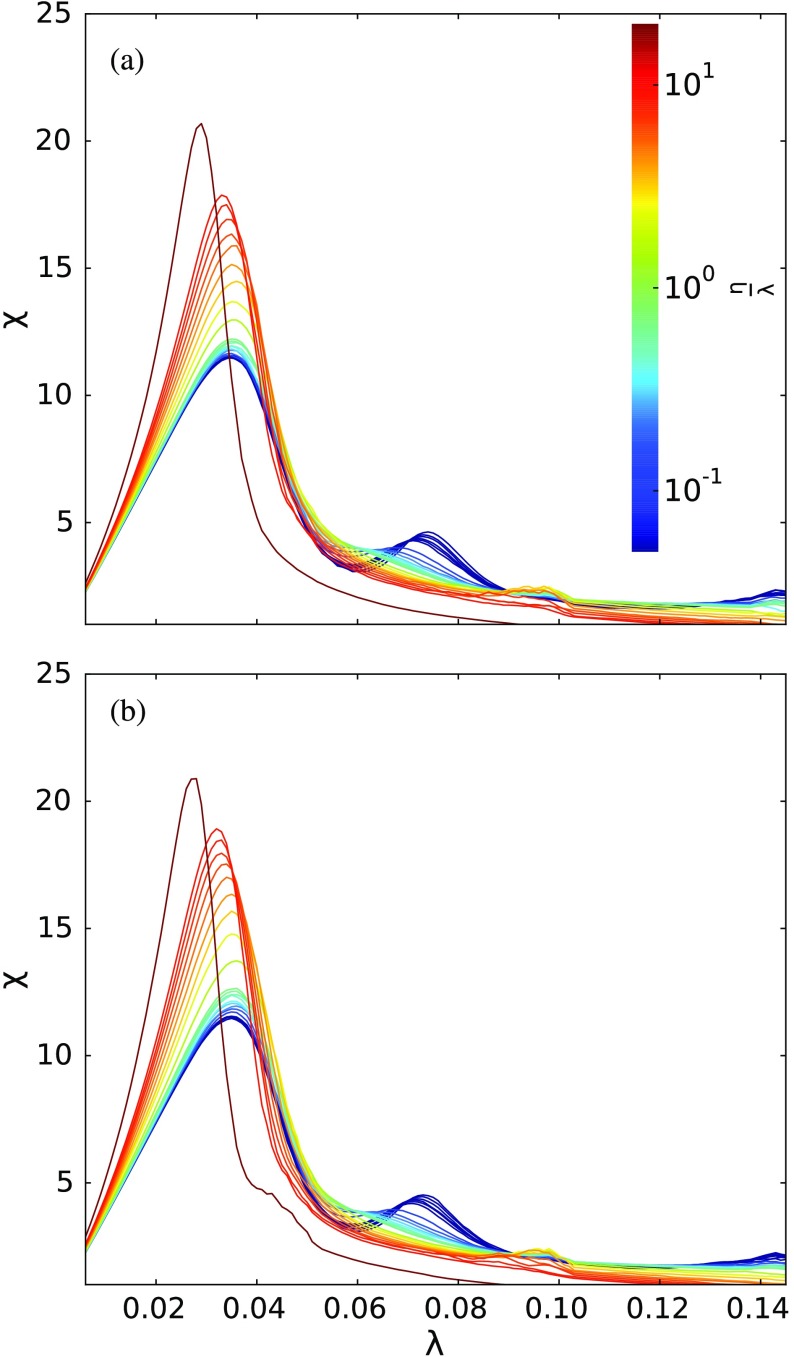
Susceptibility χ as a function of λ considering all three-layer configurations and many different ratios η/λ, which is represented by the color of the lines. The recovering rate is μ=1. The simulated values are (η/λ)=0.05, 0.06, 0.07, 0.08, 0.09, 0.1, 0.2, 0.3, 0.4, 0.5, 0.6, 0.7, 0.8, 0.9, 1.0, 2, 3, 4, 5, 6, 7, 8, 9, 10, 20.

Except for the line (2.3+2.9+2.6), all figures are similar and present similar peaks, implying that the susceptibility peaks occur approximately at the same point (for a complementary analysis, see Appendix E 3 and Fig. 18). On the other hand, the line (2.3+2.9+2.6) shows a slightly different behavior for the second peak, which is found for a larger value of λ than for the other cases. This result suggests that when the layer with the largest eigenvalue is located at the center of the line, it can effectively act as a barrier to the disease. In addition, it is verified that the extra interedges of the multiplex case do not lead to radical changes in the transition points. We remark that the susceptibility does not measure the fraction of spreaders in the steady state. Thus, despite the similarities of those curves, the phase diagrams for the incidence of the disease are different.

Coming back to what is observed for the network of layers described by the line (2.3+2.9+2.6), an interesting phenomenon arises, namely, the formation of barriers to the epidemic spreading. Since the middle layer has the lowest individual eigenvalue among the layers, it creates a barrier effect “delaying” the second transition. Moreover, we observe that this transition also vanishes for higher values of the ratio η/λ, if compared to the other cases. This can be related to the inverse participation ratio of $\Lambda_{1}$, $\mathrm{IPR}(\Lambda_{1})$, shown in Fig. 8. Note that, for the line (2.3+2.9+2.6), the contribution of the layer γ=2.6 is the lowest. As shown in Sec. V A (and in Ref. [28]), for a two-layer multiplex, the critical point of the nondominant layer shifts to a lower value of the spreading rate, which means that the outbreak takes place before it would have happened if that layer were isolated. However, here such shifting is compromised by the fact that the central layer is unable to sustain the epidemic process, acting effectively as a barrier for disease contagion. Apart from this new effect, the system behaves qualitatively similarly to the two-layer scenario.

## VII. CONCLUSIONS

In this paper, we have generalized and extended previous analyses to the case of multilayer networks. To this end, we have made use of the tensorial representation introduced in Ref. [29], which allows us to extract upper and lower bounds for the disease incidence of a SIS model and the critical points for both the SIS and the SIR dynamical processes. We have also validated our analytical insights with extensive numerical simulations, recovering results like those presented in Ref. [28] regarding the shifting of the global epidemic threshold to lower values of the spreading rate and the role of the so-called dominant layer. Furthermore, we have observed a transition in the spectra of the supra-contact tensor, from the spectra resulting from the union of the individual layers to the spectra of the network of layers. This behavior implies that other dynamics and more complex structures can also be significantly affected by the interconnected nature of the system. In addition, we have analytically characterized the phenomenon of eigenvalue crossing on the supra-contact tensor for the case of two identical layers. It is worth noticing that any dynamical process that is described by the same tensor will be affected by this.

Our main results concern the emergence and vanishing of multiple susceptibility peaks as a function of the ratio between the interlayer and intralayer spreading rates and their relation to the spectral properties of the multilayer, which also revealed the phenomenon of layerwise disease localization and, in particular, its relation to the existence of crossings or near crossings of eigenvalues. Using the QS method and Monte Carlo simulations, we have been able to precisely determine the transition points. We remark that the first susceptibility peak is a phase transition, from a disease-free state to an endemic, but layer-localized, state. On the other hand, the second peak is a transition from a layerwise localized to a delocalized state, which is not a second-order phase transition. Additionally, we have proposed an analytical approach based on the use of the inverse participation ratio to characterize such transitions as a localization phenomenon, thus also connecting with Ref. [31].

A detailed exploration of the parameter space showed that, as the ratio between the interlayer and intralayer spreading rates increases, the peaks of the susceptibility measured for the nondominant layers tend to occur at lower values of λ and vanish as η/λ increases up to a point at which only one susceptibility peak is observed, which is a true phase transition. Interestingly enough, our results point out that such a transition can take place for even lower values of λ than the inverse of the largest leading eigenvalue among all individual layers.

Finally, another important finding presented here is the opposite phenomenon, namely, the barrier effect, which happens when the susceptibility peak takes place at a larger value of λ than that expected as a consequence of the multiplex topology. Specifically, if the layers are arranged in such a way that the one with the smallest leading eigenvalue is at the center of the network of layers [for instance, as it happens for the line (2.3+2.9+2.6) configuration], then the corresponding transition could be delayed because of the barrier effect. Summarizing, our results emphasize the importance of studying multilayer systems as they are and not only as a collection of individual layers.

## Figures and Tables

**FIG. 12.**
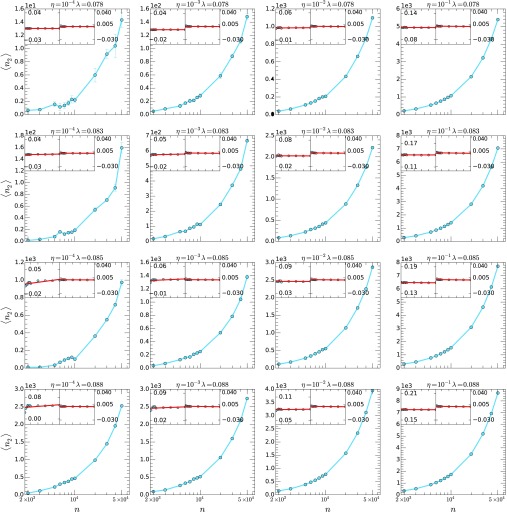
The final number of infected nodes in the second layer (with lowest individual eigenvalue) as a function of the size of the layers on the main panels. In the insets, we present the fraction of infected nodes (on the left) and the standard deviation in the steady state (on the right). The parameters used in the simulations are shown in the tile of each panel. They are a combination of the parameters λ=0.078, 0.083, 0.085, 0.088 and $\eta =10^{-4}$, $10^{-3}$, $10^{-2}$, $10^{-1}$. Furthermore, the layer sizes are $n=2\times 10^{3}$, $3\times 10^{3}$, $4\times 10^{3}$, $5\times 10^{3}$, $6\times 10^{3}$, $7\times 10^{3}$, $8\times 10^{3}$, $9\times 10^{3}$, $10^{4}$, $2\times 10^{4}$, $3\times 10^{4}$, $4\times 10^{4}$, and $5\times 10^{4}$, and m=2 in all cases.

**FIG. 13.**
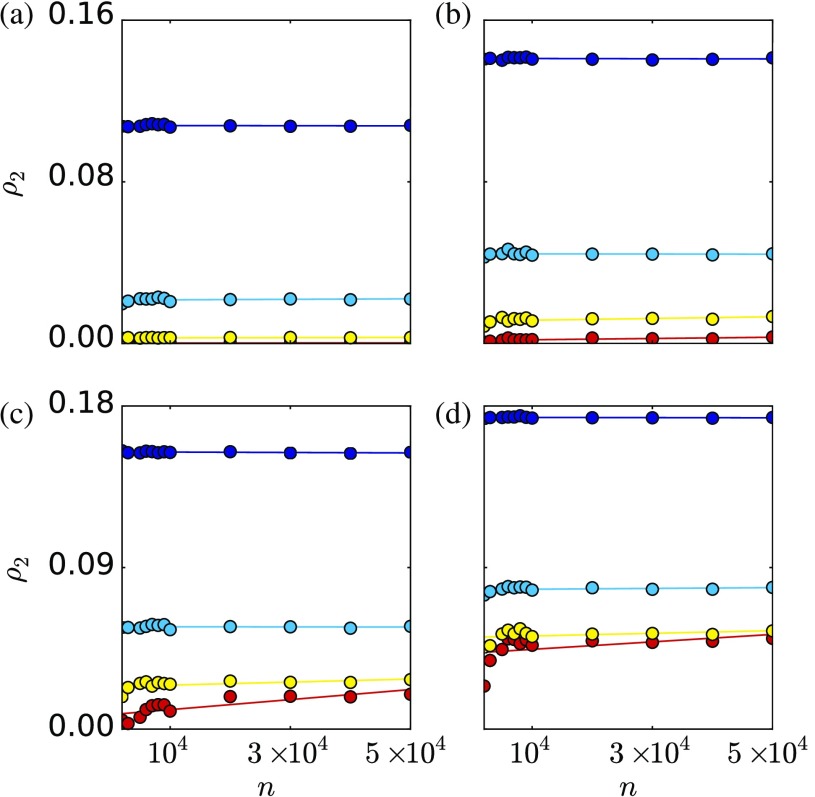
The final fraction of infected nodes in the layer with the lowest individual eigenvalue as a function of the size of the layers. The colors represent different values of η. We show λ=0.078 in panel (a), λ=0.083 in panel (b), λ=0.085 in panel (c), and λ=0.088 in panel (d). Furthermore, the layer sizes are $n=2\times 10^{3}$, $3\times 10^{3}$, $4\times 10^{3}$, $5\times 10^{3}$, $6\times 10^{3}$, $7\times 10^{3}$, $8\times 10^{3}$, $9\times 10^{3}$, $10^{4}$, $2\times 10^{4}$, $3\times 10^{4}$, $4\times 10^{4}$, and $5\times 10^{4}$, and m=2 in all cases. Each curve is the result of a parameter η; from bottom to top, $\eta =10^{-4},10^{-3},10^{-2},10^{-1}$.

**FIG. 14.**
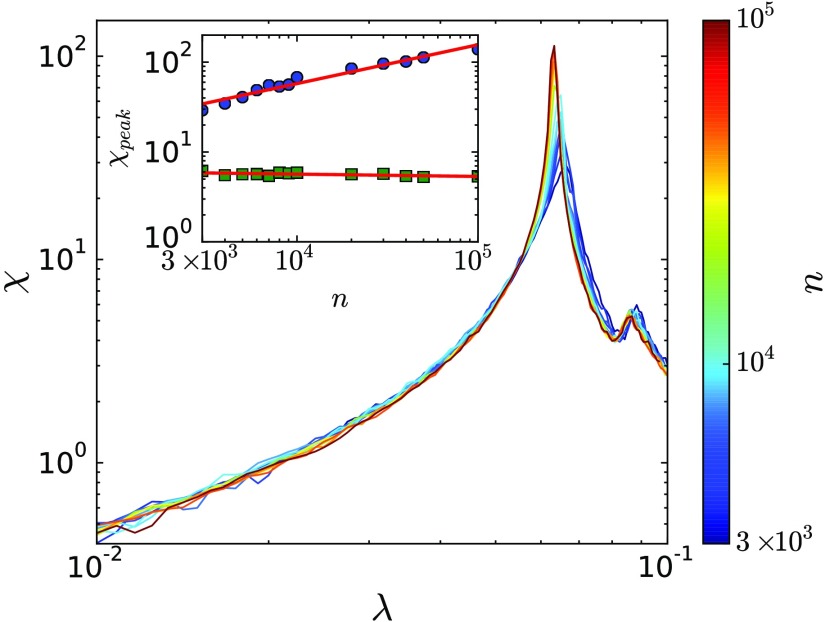
Finite-size analysis of the susceptibility. In the main panel, we have the susceptibility as a function of λ for different sizes of two-layer multiplex networks, where the first layer has ⟨k⟩=16 and the second ⟨k⟩=12. In this experiment, we fixed the ratio (η/λ)=0.01. In the inset, we show the susceptibility of the two peaks as a function of the layer size, where the blue symbols refer to the first peaks while the green symbols refer to the second peak. In addition, the red lines are a linear fitting of those points. The layer sizes evaluated are $n=3\times 10^{3}$, $4\times 10^{3}$, $5\times 10^{3}$, $6\times 10^{3}$, $7\times 10^{3}$, $8\times 10^{3}$, $9\times 10^{3}$, $10^{4}$, $2\times 10^{4}$, $3\times 10^{4}$, $4\times 10^{4}$, $5\times 10^{4}$, $10^{5}$.

**FIG. 15.**
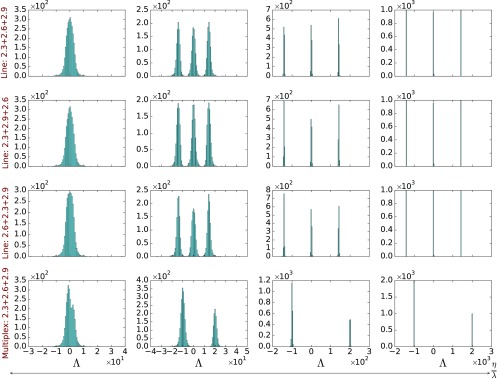
Distribution of the eigenvalues. In the rows, from top to bottom, we show the interconnected networks of lines 2.3+2.6+2.9, 2.3+2.9+2.6, 2.6+2.3+2.9 and the multiplex. In the columns, from left to right, we varied the ratios (η/λ)=1, 10, 100, and 1000, respectively. All histograms were built with 100 bins.

**FIG. 16.**
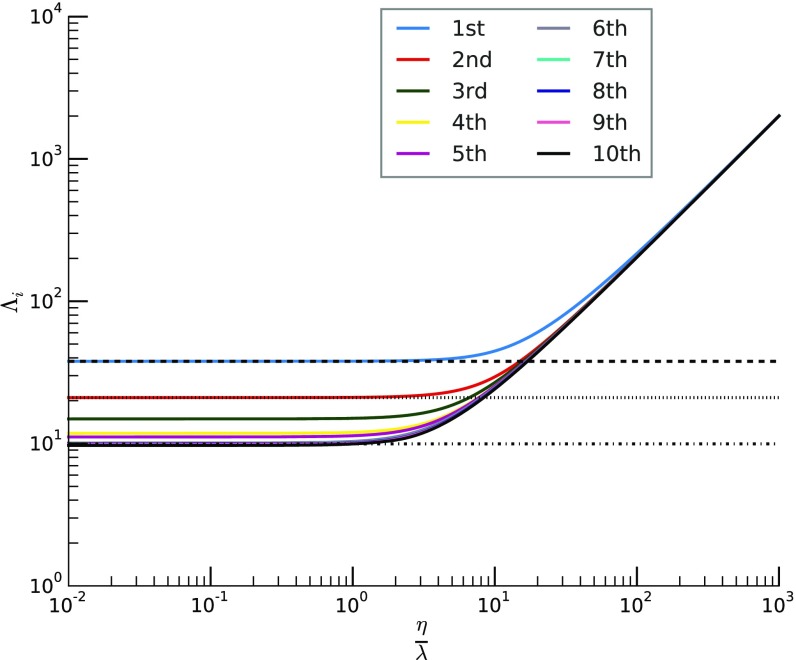
Evaluation of the eight first eigenvalues of R(λ,η) for the multiplex configuration as a function of the ratio η/λ. It is noteworthy that such a plot is visually equivalent for all the layer topologies composed of three layers. The dashed lines represent the individual-layer leading eigenvalues.

## APPENDIX A: TENSORIAL REPRESENTATION

In this appendix, we extend some important concepts of the tensorial representation. In the first subsection, we present the projections, while in the second subsection, we show the equivalence of the eigentensorial problem and the eigenvector problem of the supra-adjacency matrix. Finally, in the last subsection, we prove the relation between the tensorial projection and the matrix representation, which is fundamental to the interlacing results.

### 1. Tensorial projections

For the sake of completeness, we present other projections of multilayer networks, which are especially convenient in tensorial notation because of its compactness. Besides the adjacency tensor presented in the main text, the network of layers [30] also characterizes the topology of the system. In this reduced network representation, each node represents one layer, and the edges between them codify the number of edges connecting those two layers. Formally, we have

$$\Psi_{\tilde{\delta }}^{\tilde{\gamma }}=M_{\beta \tilde{\delta }}^{\alpha \tilde{\gamma }}U_{\alpha }^{\beta },$$ (A1)

where $\Psi_{\tilde{\delta }}^{\tilde{\gamma }}\in R^{m\times m}$. Note that such a network presents self-loops, which are weighted by the number of edges in the layer. Additionally, since we assume that the layers have the same number of nodes, the edges of the network of layers have weights equal to the number of nodes n.

Another important reduction of the multilayer network is the so-called projection [29]. Such a network aggregates all the information into one layer, including self-loops that stand for the number of layers in which a node appears. Mathematically, we have

$$P_{\beta }^{\alpha }=M_{\beta \tilde{\gamma }}^{\alpha \tilde{\delta }}U_{\tilde{\delta }}^{\tilde{\gamma }},$$ (A2)

where $P_{\beta }^{\alpha }\in R^{n\times n}$.

### 2. Eigenvalue problem

As presented in the main text, the epidemic threshold is closely related to the leading eigenvalues of the supra-contact tensor. Here, we describe the eigenvalue problem considering the tensorial representation. Such an eigenvalue problem can be generalized to the case of a rank-4 tensor, leading to

$$R_{\beta \tilde{\delta }}^{\alpha \tilde{\gamma }}f_{\alpha \tilde{\gamma }}(\Lambda )=\Lambda f_{\beta \tilde{\delta }}(\Lambda ),$$ (A3)

where Λ is an eigenvalue and $f_{\beta \tilde{\delta }}(\Lambda )$ is the corresponding eigentensor. In addition, we are assuming that the eigentensors form an orthonormal basis. Importantly, the supra-contact matrix R in Ref. [28] can be understood as a flattened version of the tensor $R_{\beta \tilde{\delta }}^{\alpha \tilde{\gamma }}(\lambda ,\eta )$. Consequently, all the results for R also apply to the tensor R. As argued in Ref. [29], that supra-adjacency matrix corresponds to unique unfolding of the fourth-order tensor m, yielding square matrices. Following this unique mapping, we have the correspondence of the eigensystems. Here, we consider that the eigenvalues are ordered as $\Lambda_{1}\geq \Lambda_{2}\geq \ldots \Lambda_{nm}$ and denote the individual-layer eigenvalues as $\Lambda_{i}^{l}$.

### 3. Proof of Eq. (8)

Consider the supra-matrix representation of a multilayer network, given by

$$A={⊕}_{\alpha }A^{\alpha }+C=\left[\begin{array}{cccc} A_{1} & C_{12} & \cdots & C_{1m}\\ C_{21} & A_{2} & \cdots & C_{2m}\\ \vdots & \vdots & \ddots & \vdots \\ C_{m1} & C_{m2} & \cdots & A_{m} \end{array}\right],$$ (A4)

where $A\in R^{nm\times nm}$, $A^{\alpha }\in R^{n\times n}$ is the adjacency matrix of the layer α∈{1,2,…m}, and C is a coupling matrix. Since we assume a multilayer network in which the layers have the same number of nodes, we have $C_{ij}=I$. We assume a partition of such a network, represented by $S\in R^{nm\times m}$, which is the characteristic matrix of such a partition, where $S_{ij}=1$ if $i\in V_{j}$ and zero otherwise, where $V_{j}$ is the network-of-layers partition.

In order to use the results of Refs. [30,34], we have to prove that the network-of-layers matrix $\bar{R}$ [30,34] is an unfolding of our tensor $\Phi_{\tilde{\delta }}^{\tilde{\gamma }}(\lambda ,\eta )$, formally given by

$$\bar{R}=\Gamma^{-1}S^{T}AS,$$ (A5)

where Γ is a diagonal matrix with normalizing constants (for more details, see Refs. [30,34]). In other words, the product AS is a summation over the blocks of the matrix A, resulting in a matrix with the degree of each node. The subsequent left product with $S^{T}$ imposes another summation, whose result is a matrix composed of the sum of all elements of the blocks. Finally, the product of $\Gamma^{-1}$ normalizes the result by $\frac{1}{n}$. Formally, we have

$$AS=\left[\begin{array}{cccc} k^{11} & k^{12} & \cdots & k^{1m}\\ k^{21} & k^{22} & \cdots & k^{2m}\\ \vdots & \vdots & \ddots & \vdots \\ k^{m1} & k^{m2} & \cdots & k^{mm} \end{array}\right],$$ (A6)

where $k^{ij}\in R^{n\times 1}$ is a vector with the number of edges emanating from each node in layer i to layer j and $AS\in R^{nm\times m}$. Then,

$$S^{T}AS=\left[\begin{array}{cccc} \sum k^{11} & \sum k^{12} & \cdots & \sum k^{1m}\\ \sum k^{21} & \sum k^{22} & \cdots & \sum k^{2m}\\ \vdots & \vdots & \ddots & \vdots \\ \sum k^{m1} & \sum k^{m2} & \cdots & \sum k^{mm} \end{array}\right],$$ (A7)

where $\sum k^{ij}\in R$ are scalars with the number of edges that connect a node in layer i to a node in layer j. Finally, the product of $\Gamma^{-1}$ introduces the average degree instead of the summation, producing the same results as Eq. (8).

## APPENDIX B: SIS MODEL ANALYSIS

In this section, we present an extension of the analysis presented in the main text regarding the SIS model. In the first subsection, we present comments on the exact model definition and its relation with the first-order approximation. Next, we present a derivation of the critical point for the first-order approximation, and finally, we present the derivation of the lower and upper bounds for such a model.

### 1. Model definition: Complementary comments

In probability theory and stochastic processes, it is common to define random variables with capital letters. However, this is the same notation usually used for tensors. In order to avoid confusion, we use bold capital letters for random variables. For instance, we define the Bernoulli random variable that defines the state of a node as $S_{\beta \overset{˜}{\delta }}$, where it assumes one of two values—zero if the node $\beta \tilde{\delta }$ is susceptible or one if it is infected. By definition, $X_{\beta \overset{˜}{\delta }}=\langle S_{\beta \overset{˜}{\delta }}\rangle$, where ⟨·⟩ is the expectation operator and $X_{\beta \tilde{\delta }}$ is the probability of the node $\beta \tilde{\delta }$ being infected.

In this way, without any assumption on the independence of random variables, the exact equation can be written as

$$\frac{d\langle S_{\beta \overset{˜}{\delta }}\rangle }{dt}=\left\langle -\mu S_{\beta \overset{˜}{\delta }}+\left(1-S_{\beta \overset{˜}{\delta }}\right)\lambda R_{\beta \overset{˜}{\delta }}^{\alpha \overset{˜}{\gamma }}(\lambda ,\eta )S_{\alpha \overset{˜}{\gamma }}\right\rangle ,$$ (B1)

where the supra-contact tensor is defined in Eq. (4). This equation can be interpreted as an exact version of the epidemic process [13]. However, without any approximation, the solution of such a problem involves $O(2^{nm})$ equations since we have to write the expressions for the expectation for all the products. The first-order approximation consists of $\langle S_{\beta \overset{˜}{\delta }}S_{\alpha \tilde{\gamma }}\rangle \approx \langle S_{\beta \overset{˜}{\delta }}\rangle \langle S_{\alpha \tilde{\gamma }}\rangle =X_{\beta \tilde{\delta }}X_{\alpha \tilde{\gamma }}$. Such an approximation is shown in Eq. (3). Interestingly, observe that Eq. (B1) can be written in terms of the covariance, defined as $\mathrm{Cov}[S_{\beta \overset{˜}{\delta }},S_{\alpha \overset{˜}{\gamma }}]=\langle S_{\beta \overset{˜}{\delta }}S_{\alpha \overset{˜}{\gamma }}\rangle -\langle S_{\beta \overset{˜}{\delta }}\rangle \langle S_{\alpha \overset{˜}{\gamma }}\rangle$. Consequently, isolating the probability of the product and substituting it in Eq. (B1), we find, by inspection, that the error is given by $\mathrm{Cov}[S_{\beta \overset{˜}{\delta }},S_{\alpha \overset{˜}{\gamma }}]$, which is assumed to be zero. In Ref. [39], the authors observed this relation and proposed an accuracy criteria for monoplex networks.

### 2. Epidemic threshold

An important concept for dynamical systems that present an absorbing state and an active phase is the critical point. Considering the SIS process, below this point, the system is inactive and the disease tends to disappear. On the other hand, above this point, we have the active phase, where the disease is present in a fraction of the population. Assuming μ>0 and that the dynamics has reached the steady state, $[dX_{\beta \overset{˜}{\delta }}/(dt)]=0$, we can write Eq. (3) as

$$\frac{X_{\beta \tilde{\delta }}^{\infty }}{1-X_{\beta \tilde{\delta }}^{\infty }}=\left(\frac{\lambda }{\mu }\right)R_{\beta \tilde{\delta }}^{\alpha \tilde{\gamma }}X_{\alpha \tilde{\gamma }}^{\infty }.$$ (B2)

Expanding the left-hand term following the geometrical series, where $[X_{\beta \overset{˜}{\delta }}^{\infty }/(1-X_{\beta \overset{˜}{\delta }}^{\infty })]=\overset{\infty }{\underset{k=1}{\sum }}{\left(X_{\beta \overset{˜}{\delta }}^{\infty }\right)}^{k}$ for $X_{\beta \tilde{\delta }}^{\infty }<1$, we obtain

$$\left(\frac{\mu }{\lambda }\right)\sum_{k=1}^{\infty }{\left(X_{\beta \tilde{\delta }}^{\infty }\right)}^{k}=R_{\beta \tilde{\delta }}^{\alpha \tilde{\gamma }}(\lambda ,\eta )X_{\alpha \tilde{\gamma }}^{\infty }.$$ (B3)

In addition, similarly to Ref. [13], suppose $X_{\beta \tilde{\delta }}^{\infty }=\varepsilon f_{\beta \tilde{\delta }}$, where ε is an arbitrary small constant and $f_{\beta \tilde{\delta }}\geq 0$. Substituting in Eq. (B3) and dividing by ε, we have

$$R_{\beta \tilde{\delta }}^{\alpha \tilde{\gamma }}(\lambda ,\eta )f_{\alpha \tilde{\gamma }}=\left(\frac{\mu }{\lambda }\right)f_{\beta \tilde{\delta }}+\varepsilon \left(\frac{\mu }{\lambda }\right){\left(f_{\beta \tilde{\delta }}\right)}^{2}+O(\varepsilon^{2}).$$ (B4)

Considering a sufficiently small ε>0, this expression reduces to the eigentensor equation

$$R_{\beta \tilde{\delta }}^{\alpha \tilde{\gamma }}(\lambda ,\eta )f_{\alpha \tilde{\gamma }}=\left(\frac{\mu }{\lambda }\right)f_{\beta \tilde{\delta }},$$ (B5)

leading to the critical point

$${\left(\frac{\mu }{\lambda }\right)}_{c}=\Lambda_{1},$$ (B6)

where $\Lambda_{1}$ is the largest eigenvalue of R, which is the same as the largest eigenvalue of R in Ref. [28].

### 3. Upper and lower bounds for the steady state

In order to obtain some bounds for the epidemic incidence, consider the steady state, where $[dX_{\beta \overset{˜}{\delta }}/(dt)]=\;0$. For a monolayer system, those bounds were calculated in Ref. [13]. We consider a multilayer network without self-loops and denote the steady state of each node as $X_{\beta \tilde{\delta }}^{\infty }$. Then, imposing $[dX_{\beta \overset{˜}{\delta }}/(dt)]=0$ in Eq. (3), we have

Xβδ˜∞=λRβδ˜αγ˜(λ,η)Xαγ˜∞λRβδ˜αγ˜(λ,η)Xαγ˜∞+μ=1-1λμRβδ˜αγ˜(λ,η)Xαγ˜∞+1. (B7)

The value of $X_{\beta \tilde{\delta }}^{\infty }$ is then obtained by iterating the above equation from an initial value, until convergence. Upper and lower bounds can be obtained by considering only the first iteration of Eq. (B7). For the upper bound, we have

$$X_{\beta \tilde{\delta }}^{\infty }\leq 1-\frac{1}{\left(\frac{\lambda }{\mu }\right)d_{\beta \tilde{\delta }}+1},$$ (B8)

where

$$d_{\beta \overset{˜}{\delta }}=R_{\beta \overset{˜}{\delta }}^{\alpha \overset{˜}{\gamma }}(\lambda ,\eta )U_{\alpha \overset{˜}{\gamma }}\;=M_{\beta \overset{˜}{\gamma }}^{\alpha \overset{˜}{\xi }}E_{\overset{˜}{\xi }}^{\overset{˜}{\gamma }}(\overset{˜}{\delta }\overset{˜}{\delta })u_{\alpha }+\frac{\eta }{\lambda }M_{\nu \overset{˜}{\delta }}^{\xi \overset{˜}{\gamma }}E_{\xi }^{\nu }(\beta \beta )u_{\overset{˜}{\gamma }}.$$ (B9)

Noticeably, there are two different contributions to the upper bound coming from intralayer and interlayer connectivity. Both of them tend to increase the probability of a node being infected. Furthermore, the higher the degree, the higher this upper bound. On the other hand, for the lower bound, let us denote $\mathrm{Min}\{X_{\beta \tilde{\delta }}^{\infty }\}=X^{\min}$. Then, substituting $X^{\min}$ in Eq. (B7), we have

$$X^{\min}\geq 1-\frac{1}{\frac{\lambda }{\mu }R_{\beta \tilde{\delta }}^{\alpha \tilde{\gamma }}(\lambda ,\eta )U_{\alpha \tilde{\gamma }}X^{\min}+1}.$$ (B10)

Denoting $\mathrm{Min}\{d_{\beta \tilde{\delta }}\}=d^{\min}$, we obtain

$$X^{\min}\geq 1-\frac{1}{\left(\frac{\lambda }{\mu }\right)d^{\min}},$$ (B11)

which can be inserted into Eq. (B7) to give

$$X_{\beta \tilde{\delta }}^{\infty }\geq X^{\min}\geq 1-\frac{1}{1+\frac{d_{\beta \tilde{\delta }}}{d^{\min}}\left[\left(\frac{\lambda }{\mu }\right)d^{\min}-1\right]}.$$ (B12)

Finally, combining Eqs. (B8) and (B12), the bounds of Eq. (3) are

$$1-\frac{1}{1+\frac{d_{\beta \tilde{\delta }}}{d^{\min}}\left[\left(\frac{\lambda }{\mu }\right)d^{\min}-1\right]}\leq X_{\beta \tilde{\delta }}^{\infty }\leq 1-\frac{1}{\left(\frac{\lambda }{\mu }\right)d_{\beta \tilde{\delta }}+1}.$$ (B13)

## APPENDIX C: SIR MODEL

Aside from the SIS epidemic model, we can also consider the SIR model. In contract with the SIS model, which has just one absorbing state (inactive), the SIR model has many absorbing states. In fact, considering an infinite population, we have an infinite number of absorbing states.

### 1. Model definition

We introduce the recovered and susceptible states, here denoted by $Y_{\beta \tilde{\delta }}$ and $Z_{\beta \tilde{\delta }}$, respectively. Then, using a similar notation as in the latter section and associating Poisson processes to nodes and edges, we have the dynamical set of equations

$$\frac{dX_{\beta \overset{˜}{\delta }}}{dt}=-\mu X_{\beta \overset{˜}{\delta }}+Z_{\beta \overset{˜}{\delta }}\lambda R_{\beta \overset{˜}{\delta }}^{\alpha \overset{˜}{\gamma }}(\lambda ,\eta )X_{\alpha \overset{˜}{\gamma }},\;\frac{dY_{\beta \overset{˜}{\delta }}}{dt}=\mu X_{\beta \overset{˜}{\delta }},\;\frac{dZ_{\beta \overset{˜}{\delta }}}{dt}=-Z_{\beta \overset{˜}{\delta }}\lambda R_{\beta \overset{˜}{\delta }}^{\alpha \overset{˜}{\gamma }}(\lambda ,\eta )X_{\alpha \overset{˜}{\gamma }}.$$ (C1)

Note that the Poisson process on the nodes correspond to the recovering mechanism, whereas on the edges they correspond to the spreading.

### 2. Epidemic threshold

Since there is no dynamic steady state in the SIR model, the epidemic threshold has a different interpretation from that of the SIS model. Above the threshold, the total number of recovered individuals reaches a finite fraction of the population, when the dynamics starts with a small fraction of infected individuals. Formally, the initial conditions are as follows: $X_{\beta \overset{˜}{\delta }}(0)=(c/nm)$, $Y_{\beta \tilde{\delta }}(0)=0$, and $Z_{\beta \overset{˜}{\delta }}(0)=1-(c/nm)$, where c is a small constant, c≪nm. Neglecting higher-order terms, we have

$$\frac{dX_{\beta \tilde{\delta }}}{dt}=-\mu X_{\beta \tilde{\delta }}+\lambda R_{\beta \tilde{\delta }}^{\alpha \tilde{\gamma }}(\lambda ,\eta )X_{\alpha \tilde{\gamma }}.$$ (C2)

After a proper factorization,

$$\frac{dX_{\beta \tilde{\delta }}}{dt}=\lambda \left(R_{\beta \tilde{\delta }}^{\alpha \tilde{\gamma }}(\lambda ,\eta )-\frac{\mu }{\lambda }\delta_{\beta \tilde{\delta }}^{\alpha \tilde{\gamma }}\right)X_{\alpha \tilde{\gamma }},$$ (C3)

where $\delta_{\beta \tilde{\delta }}^{\alpha \tilde{\gamma }}$ is a tensor analogous to the identity matrix, whose elements are 1 if the indices are the same. The epidemic threshold is as in Eq. (6), which is the critical value for both SIR and SIS dynamics.

## APPENDIX D: TWO-LAYER MULTIPLEX SYSTEMS

In this section, we present some complementary analyses for the two-layer multiplex case. Here, we focus on some spectral aspects of such systems, mainly on the eigenvalue crossing and near-crossing phenomenon, presented in the first subsection, and additionally on the second susceptibility peak using a finite-size analysis. These results are presented in the second subsection and are complementary to Sec. V D in the main text.

### 1. Spectral aspects

In this section, we focus on the spectral analysis of the tensor R(λ,η) as a function of the ratio η/λ. First, we present an analytical approach to the problem of eigenvalue crossings; then, we focus on three special cases in increasing order of complexity. First is the identical case, where both layers are exactly the same. Thus, there is a high correlation between the degree in each layer, presented in b. Next we focus on the nonidentical case, where both layers present the same degree distribution, but different configurations. The case of two different layer structures, considering that their leading eigenvalues are spaced, was presented in the main text.

#### a. Eigenvalue crossing

Let us analyze the spectra of a simple setup: multiplex networks composed of l identical layers. Such a class of networks provides insights about the spectral behavior as a function of (η/λ). Although they are not very realistic *a priori*, there are situations in which this representation is helpful: For instance, in the context of disease contagion, one might think of a multistrain disease in which each strain propagates in a different layer, allowing co-infection of the host population.

The adjacency tensor can be written as

$$R_{\beta \tilde{\delta }}^{\alpha \tilde{\gamma }}(\lambda ,\eta )=A_{\beta }^{\alpha }\delta_{\tilde{\delta }}^{\tilde{\gamma }}+\frac{\eta }{\lambda }\delta_{\beta }^{\alpha }K_{\tilde{\delta }}^{\tilde{\gamma }},$$ (D1)

where $A_{\beta }^{\alpha }$ is the rank-2 layer adjacency tensor, $K_{\tilde{\gamma }}^{\tilde{\delta }}$ is the adjacency tensor of the network of layers, which is a complete graph on the multiplex case, and $\delta_{\beta }^{\alpha }$ is the Kronecker delta. Observe that the sum of two Kronecker products, $\overset{¯}{A}=I_{m}⊗A+(\eta /\lambda )K_{m}⊗I_{n}$, where $I_{n}$ is the identity matrix of size n and $K_{m}$ is the adjacency matrix of the complete graph with m nodes, is the unfolding of the adjacency tensor in this case. In this way, the eigenvalue problem can be written as

$$R_{\beta \tilde{\delta }}^{\alpha \tilde{\gamma }}f_{\alpha \tilde{\gamma }}=A_{\beta }^{\alpha }\delta_{\tilde{\delta }}^{\tilde{\gamma }}f_{\alpha \tilde{\gamma }}+\frac{\eta }{\lambda }\delta_{\beta }^{\alpha }K_{\tilde{\delta }}^{\tilde{\gamma }}f_{\alpha \tilde{\gamma }},$$ (D2)

where the sums of the eigenvalues of A, $\Lambda_{i}^{l}$ and K, $\mu_{i}$ are also eigenvalues of the adjacency tensor; hence, $R_{\beta \overset{˜}{\delta }}^{\alpha \overset{˜}{\gamma }}f_{\alpha \overset{˜}{\gamma }}=\left[\Lambda_{i}^{l}+(\eta /\lambda )\mu_{j}\right]f_{\alpha \overset{˜}{\gamma }}$, i=1,2,…,n and j=1,2,…,m. Then,

$$\left(\Lambda_{i}^{l}+\frac{\eta }{\lambda }\mu_{j}\right)=\left(\Lambda_{k}^{l}+\frac{\eta }{\lambda }\mu_{s}\right).$$ (D3)

The eigenvalues of the complete graph are $\mu_{1}=m-1$ and $\mu_{i}=-1$, ∀  i>1, yielding

$$\frac{\eta }{\lambda }=\frac{\Lambda_{k}^{l}-\Lambda_{i}^{l}}{m},$$ (D4)

which imposes crossings on the eigenvalues of the adjacency tensor for identical layers since (η/λ) is a continuous parameter.

#### b. Identical layers

Consider a multiplex network made up of two layers with the same configuration. Each layer of the multiplex is a network composed of n=1000, ⟨k⟩≈6, $\Lambda^{l}=14.34$, with degree distribution $P(k)∼k^{-2.7}$. Aside from the intra-edge configuration, we also impose that interedges connect a node with its counterpart on the other layer; i.e., every node has the same intradegree on all layers. Such a constraint imposes a high correlation between the degrees in each layer.

Figure 10 shows the spectral behavior of such a multiplex as a function of the parameter (η/λ). In the top panel, we present the inverse participation ratio of the first three eigenvalues, while in the bottom panel, we plot the first ten eigenvalues. When the ratio (η/λ)=0, the eigenvalues have multiplicity 2, as can be seen on the left side of the bottom panel (approximately, since the figure starts from $10^{-2}$). More importantly, those eigenvalues tend to behave differently: One increases, while the other tends to decrease. This behavior leads to the eigenvalue crossing (see Appendix 1 a). The inset of the bottom panel zooms out on the region where the crossing takes place. Note that the eigenvalues cross at the same value for which the inverse participation ratio shows an abrupt change. Indeed, the jump in the IPR(Λ) has its roots in the interchange of the eigenvectors associated with each of the eigenvalues that are crossing. Moreover, we stress that the abrupt change observed for IPR(Λ) is always present in such scenarios, but it could be either from the lower to the higher values or vice versa depending on the structure of the layers.

#### c. Similar layers

In addition to the identical case, we have also considered a multiplex network composed of two layers with the same degree distribution (i.e., the same degree sequence), with $P(k)∼k^{-2.7}$, but different random realizations of the configuration model. Furthermore, the interedges follow the same rule as before, connecting nodes with their counterparts in the other layer, assuring that every node has the same intradegree in all layers. Each layer of the multiplex network is composed of n=1000 and ⟨k⟩≈6. Since each layer is a different realization of the configuration model, both present a slightly different leading eigenvalue—the first $\Lambda_{1}^{1}=15.21$ and the second $\Lambda_{1}^{2}=14.34$.

Figure 11 shows the spectral behavior of such a multiplex in terms of the largest eigenvalues (in the bottom panel) and the IPR(Λ) (in the top panel). Here, in addition to the global inverse participation ratio, we also present the contribution of each layer to this measure. Such an analysis is meaningless in the identical case since the contribution is the same. As shown in the figure, we observe that for small values of η/λ, in regard to the first eigenvalue, the system is localized on the first layer and delocalized on the second. On the other hand, the picture changes when we focus on the second eigenvalue, as it is localized in the second layer but delocalized in the first. For larger values of η/λ, both layers contribute equally to IPR(Λ). Analogously to the identical case, there is a change in $\mathrm{IPR}(\Lambda_{2})$, which seems to be related to the changes in $\Lambda_{2}$, as one can see in the bottom panel and in the inset. Note that for this case, there is no crossing; i.e., the eigenvalues avoid the crossing—also referred to as near crossing.

### 2. Finite-size analysis

In this section, we analyze the behavior of a two-layer multiplex network at the steady state, considering different sizes. Such a multiplex was built considering two Erdös-Rényi networks with a fixed mean degree. As mentioned in the main text, we chose these networks because their epidemic thresholds do not vanish at the thermodynamic limit, which contrasts with the scale-free networks. In this way, we have a well-defined critical point that can be precisely tuned regardless of the network size. Following the usual convention in the complex network literature, the first susceptibility peak observed in our experiments can be classified as a critical point of a phase transition. In such a point, the dynamics goes from a disease-free state to an endemic state. However, the second susceptibility peak cannot be classified as a second-order phase transition since the disease is already in an endemic state. Although it cannot be considered as a critical point, before the second susceptibility peak, most of the events take place in only one layer (the one with the largest individual eigenvalue), while after this point, both layers are active and spreading the disease.

Similarly to the experiments shown in Sec. V D, here we run the continuous simulation 50 times and perform a moving average filter over a sampling of the original time series, resulting in $5\times 10^{4}$ points. The simulations are run up to $t=10^{3}$. Note that for continuous simulations, the number of points can vary from one run to another. The steady-state statistics are estimated for t≥950 or, in other words, the last 50 time units. In contrast with the main text, here we are interested in comparing results for different network sizes: $n=2\times 10^{3}$, $3\times 10^{3}$, $4\times 10^{3}$, $5\times 10^{3}$, $6\times 10^{3}$, $7\times 10^{3}$, $8\times 10^{3}$, $9\times 10^{3}$, $10^{4}$, $2\times 10^{4}$, $3\times 10^{4}$, $4\times 10^{4}$, and $5\times 10^{4}$, and m=2 in all cases. In addition, we considered the mean degree as ⟨k⟩=16 for the first layer and ⟨k⟩=12 for the second. We expect that the second susceptibility peak appears near the epidemic threshold of the second layer individually, i.e., λ≈0.083.

Figure 12 presents the number of infected nodes in the steady state on the layer with the lowest individual eigenvalue as a function of the size of the layers and a combination of the parameters λ=0.078, 0.083, 0.085, 0.088 (near the individual critical point of the second layer) and $\eta =10^{-4}$, $10^{-3}$, $10^{-2}$, $10^{-1}$. In addition, in the insets, we have the information about the average fraction (left inset in each panel) and its fluctuations, measured by the standard deviation (right inset in each panel). The straight lines in red were obtained by a least-squares regression method.

We observe an approximately linear behavior of the number of infected nodes in the second layer as a function of the number of nodes in such a layer (see the main panels of Fig. 12). Consequently, the fraction $\rho_{2}$ also presents a linear trend (see the left inset in each panel of Fig. 12). In fact, it presents a flat pattern, i.e., approximately constant. In addition, the number of infected nodes is always larger than zero since it is not a disease-free state. Furthermore, we also observed that the fluctuations tend to be very low (see the right inset in each panel of Fig. 12). Regarding the fluctuations, it is noteworthy that, in a phase transition, they tend to diverge, which does not happen in our analysis, thus also ruling out a second-order phase transition as far as we are concerned. We also note that fluctuations are slightly higher for lower spreading rates, as can be seen by the error bars for λ=0.078, which is explained by the delocalization of our system.

Furthermore, in Fig. 13, we present the comparison of steady-state fractions. In each panel, we fix a value of λ and compare different values of η. We emphasize the influence of η on the final fraction of infected nodes in the second layer. Note that for $\eta =10^{-4}$ and small networks, the behavior exhibits a growing trend. This is due to the fact that for networks with $n<10^{4}$, the contribution of the first layer can be effectively neglected. In fact, observe that for $n\geq 10^{4}$ the fraction of infected nodes in the second layer follows a flat pattern [see Figs. 13(c) and 13(d)]. Finally, in Fig. 14, we present a finite-size analysis of the susceptibility for different sizes, ranging from $n=3\times 10^{3}$ to $n=10^{5}$ and m=2 layers. Each curve was obtained using the QS algorithm, with which we simulated 120 points from $\lambda =10^{-2}$ to $\lambda =10^{-1}$. In this experiment, we fixed the ratio (η/λ)=0.01. Additionally, we also used a moving average filter with two points for visualization purposes. In the inset, we show the scaling of the susceptibility corresponding to the two peaks. The positive slope for the first peak indicates that it divergences as the system size goes to infinity, thus evidencing the phase transition. On the other hand, the curve for the case of the second peak is flat no matter what the value of the system size is, indicating that in contrast to the behavior observed for the first peak, in this case there is no divergence in the thermodynamic limit and the peak does not vanish.

## APPENDIX E: THREE-LAYER INTERCONNECTED SYSTEMS: COMPLEMENTARY ANALYSIS

In this section, we study the introduction of a third layer, which increases the complexity of the system, allowing four different network layer configurations: the line, which has three different configurations depending on the position of the layers, and the triangle, which is also a multiplex. This section is organized as follows: In the first subsection, we perform the spectral analysis of the adjacency tensor as a function of the parameter η/λ, showing that as we increase this parameter, the spectral distribution tends to the spectra of the network of layers, which is explained by interlacing theorems. Next, in Appendixes E 2 and E 3, we show the complementary results of localization and susceptibility analysis, respectively.

### 1. Spectral analysis

Since the epidemic process is described through the supra-adjacency tensor R(λ,η), its spectral properties give us some insights about the whole process, especially about the critical properties of the systems under analysis. Moreover, as the structure of the network of layers is not trivial anymore, we find important differences regarding the spectra of such tensors for the different topologies of the network of layers.

Figure 15 shows the spectrum of the four configurations of networks when varying the ratio (η/λ)=1, 10, 100, and 1000. Observe that we do not show the ratio (η/λ)=0 since it is just the union of the individual-layer spectrum. For (η/λ)=1, the four configurations are very similar, especially the line graphs. In such a case, the interlayer edges are treated in the same way as the intralayer ones. In other words, they are ignored, and the network can be interpreted as a monoplex network. As the spreading ratio increases, the spectrum tends to be clustered near the values of the eigenvalues of the network of layers. Such a spectrum was analytically calculated in Sec. II and shown in Table I in the main text.

Regarding the triangle configuration, the clustering of the spectrum as η/λ increases is even clear. Triangles present the lowest eigenvalue with multiplicity two. On the extreme case of (η/λ)≫1 (see Fig. 15), we have 2/3 of the values near the left extreme value, while 1/3 of them are near the leading eigenvalue. On the other hand, for the line configurations, the frequencies of the eigenvalue distribution is related to the position of the central layer. However, in the limiting cases, such differences are reduced. This pattern is naturally related to the increase of the spreading ratio: When η/λ increases, so does the role of the interlayer edges relative to the intralayer ones. Consequently, the structure of the network of layers imposes itself more strongly on the eigenvalues of the entire interconnected structure. This comes as a consequence of the interlacing theorems shown in Sec. III A in the main text.

Our findings can be related to the structural transition shown in Ref. [40], where the authors evaluated the supra-Laplacian matrix as a function of the interlayer weights. Their main result is an abrupt structural transition from a decoupled regime, where the layers seem to be independent, to a coupled regime, where the layers behave as one single system. Here, we are interested in the supra-adjacency tensor; however, we found a similar phenomenological behavior and a structural change of the system as a function of the interlayer weights, which in our case are determined by a dynamical process.

### 2. Localization in interconnected networks

Complementary to the results presented in Sec. VI, here we present results for the lines (2.3+2.6+2.6) and (2.6+2.3+2.6). Similarly, the experiments here are conducted in terms of the inverse participation ratio, as was done for the two-layer multiplex case.

Figure 16 shows the tenth largest eigenvalues of the three-layer multiplex case. The dashed lines represent the leading eigenvalue of each layer. Note that the leading eigenvalue of the layer with $P(k)∼k^{-2.9}$ is the seventh largest in the network spectrum when (η/λ)=0. We observe that there are no crossings in the observed eigenvalues, which is an expected result since the layers have different structures. Furthermore, it is important to remark that all networks of layers evaluated also show similar qualitative behaviors. The topology of the network of layers does not lead to qualitative differences in the dependence of $\Lambda_{i}$ on η/λ for the first ten eigenvalues. We also notice that, although it is only an approximation, perturbation theory would be valid roughly up to $\frac{\eta }{\lambda }≲10$.

Figures 8 and 17 show the $\mathrm{IPR}(\Lambda_{1})$. In the main panel, we present the individual contribution of each layer, while in the insets, we have the total $\mathrm{IPR}(\Lambda_{1})$. As mentioned in the main text, the first eigenvalue is usually enough to analyze the localization as a first-order approximation. Here, we observe that the layer with the largest eigenvalue dominates the dynamics. In addition, note the similarities between the multiplex and the line configuration (2.6+2.3+2.6), where the nondominant layers behave similarly. This is because for small values of η/λ, the effect of the extra edge in the network of layers (closing the triangle) is of order $\eta^{2}$, thus the similar behavior observed comparing panel (b) of Figs. 8 and 17 for the two configurations. As η/λ grows, the symmetry in the node-aligned multiplex dominates the eigenvector structure, and the contributions of all layers are comparable. As we show next, the different contributions of the layers to the total $\mathrm{IPR}(\Lambda_{1})$ are at the root of the multiple susceptibility peaks observed.

Complementing and reinforcing the analysis of Section VI, comparing the different line configurations of the network of layers, we observe that the largest eigenvalue of the whole system, $\Lambda_{1}$, has its associated eigenvector localized in the dominant layer, that is, in the layer generated using γ=2.3. Depending on the position of that layer in the whole system—i.e., central or peripheral layer—the contribution of the nondominant layers to $\mathrm{IPR}(\Lambda_{1})$ varies. In particular, when the dominant layer corresponds to an extreme node of the network of layers, the contribution of the other two layers will be ordered according to the distance to the dominant one. Consequently, when the dominant layer is in the center of the network of layers, the contributions of the nondominant ones are comparable—note that in panel (b) of Fig. 17, there is no difference in the contribution to $\mathrm{IPR}(\Lambda_{1})$ for layers generated using γ=2.6 and γ=2.9.

### 3. Multiple susceptibility peaks: Additional results

Figure 18 shows the susceptibility as a function of λ for different ratios of η/λ. As observed in the main text, we also have three well-defined peaks in these curves when the ratio η/λ is small. In addition, similar to the two-layer case, such peaks tend to become less defined and vanish as the ratio η/λ increases.

Regarding the third peak, note that it is less defined than the others because the average number of infected nodes is larger in this case. Consequently, the susceptibility tends to be lower since it measures the variance in relation to the average. The comparison of Figs. 9(b) and 18 shows that there is no difference in the position of the susceptibility peaks. As mentioned in the main text, the only observed difference is the barrier effect, shown in Fig. 9(a). We also remark the similarities between the line (2.6+2.3+2.6) and the multiplex case, which emphasize the role of the central node. In that line configuration, the layer with γ=2.3 spreads its influence to both layers, since it is similar to the multiplex case, however with less intra-edges.

## References

[1] R. Pastor-Satorras, C. Castellano, P. Van Mieghem, and A. Vespignani, Epidemic Processes in Complex Networks, Rev. Mod. Phys. 87, 925 (2015). RMPHAT 0034-6861 10.1103/RevModPhys.87.925

[2] R. M. Anderson and R. M. May, Infectious Diseases of Humans Dynamics and Control (Oxford University Press, New York, 1992).

[3] A. Barrat, M. Barthlemy, and A. Vespignani, Dynamical Processes on Complex Networks (Cambridge University Press, New York, NY, 2008).

[4] M. E. J. Newman, The Structure and Function of Complex Networks, SIAM Rev. 45, 167 (2003). SIREAD 0036-1445 10.1137/S003614450342480

[5] S. Boccaletti, V. Latora, Y. Moreno, M. Chavez, and D. U. Hwang, Complex Networks: Structure and Dynamics, Phys. Rep. 424, 175 (2006). PRPLCM 0370-1573 10.1016/j.physrep.2005.10.009

[6] L. F. Costa, F. A. Rodrigues, G. Travieso, and P. R. V. Boas, Characterization of Complex Networks: A Survey of Measurements, Adv. Phys. 56, 167 (2007). ADPHAH 0001-8732 10.1080/00018730601170527

[7] R. Pastor-Satorras and A. Vespignani, Epidemic Spreading in Scale-Free Networks, Phys. Rev. Lett. 86, 3200 (2001). PRLTAO 0031-9007 10.1103/PhysRevLett.86.3200 11290142

[8] M. Boguñá and R. Pastor-Satorras, Epidemic Spreading in Correlated Complex Networks, Phys. Rev. E 66, 047104 (2002). PRESCM 1539-3755 10.1103/PhysRevE.66.047104 12443385

[9] M. Newman, Networks: An Introduction (Oxford University Press, New York, 2010).

[10] P. Van Mieghem, Epidemic Phase Transition of the SIS Type in Networks, Europhys. Lett. 97, 48004 (2012). EULEEJ 0295-5075 10.1209/0295-5075/97/48004

[11] S. C. Ferreira, C. Castellano, and R. Pastor-Satorras, Epidemic Thresholds of the Susceptible-Infected-Susceptible Model on Networks: A Comparison of Numerical and Theoretical Results, Phys. Rev. E 86, 041125 (2012). PRESCM 1539-3755 10.1103/PhysRevE.86.041125 23214547

[12] Y. Wang, D. Chakrabarti, C. Wang, and C. Faloutsos, Epidemic Spreading in Real Networks: An Eigenvalue Viewpoint, in SRDS (2003), pp. 25–34, http://ieeexplore.ieee.org/document/1238052/?reload=true.

[13] P. Van Mieghem, J. Omic, and R. Kooij, Virus Spread in Networks, IEEE/ACM Trans. Netw. 17, 1 (2009). IEANEP 1063-6692 10.1109/TNET.2008.925623

[14] S. Gómez, A. Arenas, J. Borge-Holthoefer, S. Meloni, and Y. Moreno, Discrete-Time Markov Chain Approach to Contact-Based Disease Spreading in Complex Networks, Europhys. Lett. 89, 38009 (2010). EULEEJ 0295-5075 10.1209/0295-5075/89/38009

[15] P. Holme and J. Saramäki, Temporal Networks, Phys. Rep. 519, 97 (2012). PRPLCM 0370-1573 10.1016/j.physrep.2012.03.001

[16] E. Valdano, L. Ferreri, C. Poletto, and V. Colizza, Analytical Computation of the Epidemic Threshold on Temporal Networks, Phys. Rev. X 5, 021005 (2015). PRXHAE 2160-3308 10.1103/PhysRevX.5.021005

[17] M. E. J. Newman, Threshold Effects for Two Pathogens Spreading on a Network, Phys. Rev. Lett. 95, 108701 (2005). PRLTAO 0031-9007 10.1103/PhysRevLett.95.108701 16196976

[18] A. B. Pedersen and A. Fenton, Emphasizing the Ecology in Parasite Community Ecology, Trends Ecology Evolution 22, 133 (2007). 10.1016/j.tree.2006.11.005 17137676

[19] H. J. Wearing, D. A. Vasco, Y. Huang, and P. Rohani, Understanding Host-Multipathogen Systems: Modeling the Interaction between Ecology and Immunology, in Infectious Disease Ecology: Effects of Ecosystems on Disease and of Disease on Ecosystems (Princeton University Press, Princeton, 2008), pp. 48–70.

[20] P.-A. Noël, A. Allard, L. Hébert-Dufresne, V. Marceau, and L. J. Dubé, Propagation on Networks: An Exact Alternative Perspective, Phys. Rev. E 85, 031118 (2012). PRESCM 1539-3755 10.1103/PhysRevE.85.031118 22587049

[21] C. Poletto, S. Meloni, V. Colizza, Y. Moreno, and A. Vespignani, Host Mobility Drives Pathogen Competition in Spatially Structured Populations, PLoS Comput. Biol. 9, e1003169 (2013). PCBLBG 1553-7358 10.1371/journal.pcbi.1003169 PMC3744403 23966843

[22] F. D. Sahneh and C. Scoglio, Competitive Epidemic Spreading over Arbitrary Multilayer Networks, Phys. Rev. E 89, 062817 (2014). PRESCM 1539-3755 10.1103/PhysRevE.89.062817 25019843

[23] J. Sanz, C.-Y. Xia, S. Meloni, and Y. Moreno, Dynamics of Interacting Diseases, Phys. Rev. X 4, 041005 (2014). PRXHAE 2160-3308 10.1103/PhysRevX.4.041005

[24] S. Funk, E. Gilad, C. Watkins, and V. A. A. Jansen, The Spread of Awareness and Its Impact on Epidemic Outbreaks, Proc. Natl. Acad. Sci. U.S.A. 106, 6872 (2009). PNASA6 0027-8424 10.1073/pnas.0810762106 PMC2672559 19332788

[25] S. Funk, M. Salathé, and V. A. A. Jansen, Modelling the Influence of Human Behaviour on the Spread of Infectious Diseases: A Review, J. R. Soc. Interface 7, 1247 (2010).1742-5689 10.1098/rsif.2010.0142 PMC2894894 20504800

[26] M. Sandro, P. Nicola, A. Alex, G. Sergio, M. Yamir, and V. Alessandro, Modeling Human Mobility Responses to the Large-Scale Spreading of Infectious Diseases, Sci. Rep. 1, 62 (2011). SRCEC3 2045-2322 10.1038/srep00062 PMC3216549 22355581

[27] M. Kivelä, A. Arenas, M. Barthelemy, J. P. Gleeson, Y. Moreno, and M. A. Porter, Multilayer Networks, J. Complex Netw. 2, 203 (2014).2051-1329 10.1093/comnet/cnu016

[28] E. Cozzo, R. A. Baños, S. Meloni, and Y. Moreno, Contact-Based Social Contagion in Multiplex Networks, Phys. Rev. E 88, 050801 (2013). PRESCM 1539-3755 10.1103/PhysRevE.88.050801 24329202

[29] M. De Domenico, A. Solé-Ribalta, E. Cozzo, M. Kivelä, Y. Moreno, M. A. Porter, S. Gómez, and A. Arenas, Mathematical Formulation of Multilayer Networks, Phys. Rev. X 3, 041022 (2013). PRXHAE 2160-3308 10.1103/PhysRevX.3.041022

[30] R. J. Sánchez-García, E. Cozzo, and Y. Moreno, Dimensionality Reduction and Spectral Properties of Multilayer Networks, Phys. Rev. E 89, 052815 (2014). PRESCM 1539-3755 10.1103/PhysRevE.89.052815 25353852

[31] A. V. Goltsev, S. N. Dorogovtsev, J. G. Oliveira, and J. F. F. Mendes, Localization and Spreading of Diseases in Complex Networks, Phys. Rev. Lett. 109, 128702 (2012). PRLTAO 0031-9007 10.1103/PhysRevLett.109.128702 23006000

[32] G. F. de Arruda, E. Cozzo, Y. Moreno, and F. A. Rodrigues, On Degree-Degree Correlations in Multilayer Networks, Physica (Amsterdam) 323D–324D, 5 (2016). PDNPDT 0167-2789 10.1016/j.physd.2015.11.004

[33] E. Cator and P. Van Mieghem, Nodal Infection in Markovian Susceptible-Infected-Susceptible and Susceptible-Infected-Removed Epidemics on Networks Are Non-Negatively Correlated, Phys. Rev. E 89, 052802 (2014). PRESCM 1539-3755 10.1103/PhysRevE.89.052802 25353839

[34] E. Cozzo, G. F. Arruda, F. A. Rodrigues, and Y. Moreno, Interconnected Networks (Springer International Publishing, New York, 2016), Chap. “Multilayer Networks: Metrics and Spectral Properties,” pp. 17–35.

[35] A. S. Mata and S. C. Ferreira, Multiple transitions of the susceptible-infected-susceptible epidemic model on complex networks, Phys. Rev. E 91, 012816 (2015). PRESCM 1539-3755 10.1103/PhysRevE.91.012816 25679666

[36] J. R. Dormand and P. J. Prince, A Family of Embedded Runge-Kutta Formulae, J. Comput. Appl. Math. 6, 19 (1980). JCAMDI 0377-0427 10.1016/0771-050X(80)90013-3

[37] F. Viger and M. Latapy, Efficient and Simple Generation of Random Simple Connected Graphs with Prescribed Degree Sequence, in Proceedings of the 11th Annual International Conference on Computing and Combinatorics, COCOON’05 (Springer-Verlag, Berlin, Heidelberg, 2005), pp. 440–449.

[38] P. Van Mieghem and E. Cator, Epidemics in Networks with Nodal Self-Infection and the Epidemic Threshold, Phys. Rev. E 86, 016116 (2012). PRESCM 1539-3755 10.1103/PhysRevE.86.016116 23005500

[39] P. Van Mieghem and R. van de Bovenkamp, Accuracy Criterion for the Mean-Field Approximation in Susceptible-Infected-Susceptible Epidemics on Networks, Phys. Rev. E 91, 032812 (2015). PRESCM 1539-3755 10.1103/PhysRevE.91.032812 25871162

[40] F. Radicchi and A. Arenas, Abrupt Transition in the Structural Formation of Interconnected Networks, Nat. Phys. 9, 717 (2013). NPAHAX 1745-2473 10.1038/nphys2761

